# Supplementary material for: Repeated nuclear translocations underlie photoreceptor positioning and lamination of the outer nuclear layer in the mammalian retina
Source: Cell Rep. 2021 Aug 3;36(5):109461. doi: 10.1016/j.celrep.2021.109461 (PMC8356022; doi:10.1016/j.celrep.2021.109461)
Supplement: Document S1. Figures S1–S5 [file mmc1.pdf]

Cell Reports, Volume *36*

Supplemental information

Repeated nuclear translocations underlie photoreceptor positioning and lamination of the outer nuclear layer in the mammalian retina

Nozie D. Aghaizu, Katherine M. Warre-Cornish, Martha R. Robinson, Paul V. Waldron, Ryea N. Maswood, Alexander J. Smith, Robin R. Ali, and Rachael A. Pearson

	DMSO	Demecolcine	Blebbistatin	Ciliobrevin D	*shCtrl/DsRed*	*shDync1h1/EGFP*
Normalised # basal events	3.1 ± 0.5	3.4 ± 0.6	2.7 ± 0.7	2.6 ± 1.3	3.4 ± 0.6	2.4 ± 0.3
Normalised # apical events	0.3 ± 0.1	0.2 ± 0.1	0.4 ± 0.2	0.0 ± 0.0 **	0.4 ± 0.0	0.1 ± 0.1 **

Table S1. Frequency of rapid apically- and basally-directed events.

Related to Figure 1-5.

Normalised number of observed rapid apically- and basally-directed events per 1000 total recording minutes in DMSO control or drug exposed live P3 retinal explants subjected to time lapse microscopy. Event count was normalised by cumulative recording minutes (see Methods). *shCtrl/DsRed* and *shDync1h1/EGFP* data were obtained from live imaged *Nrl.Cre*$^{+/-}$ retinae that had previously been electroporated at P1 and cultured for 6DIV. $^{**}p < 0.01$; unpaired *t* test

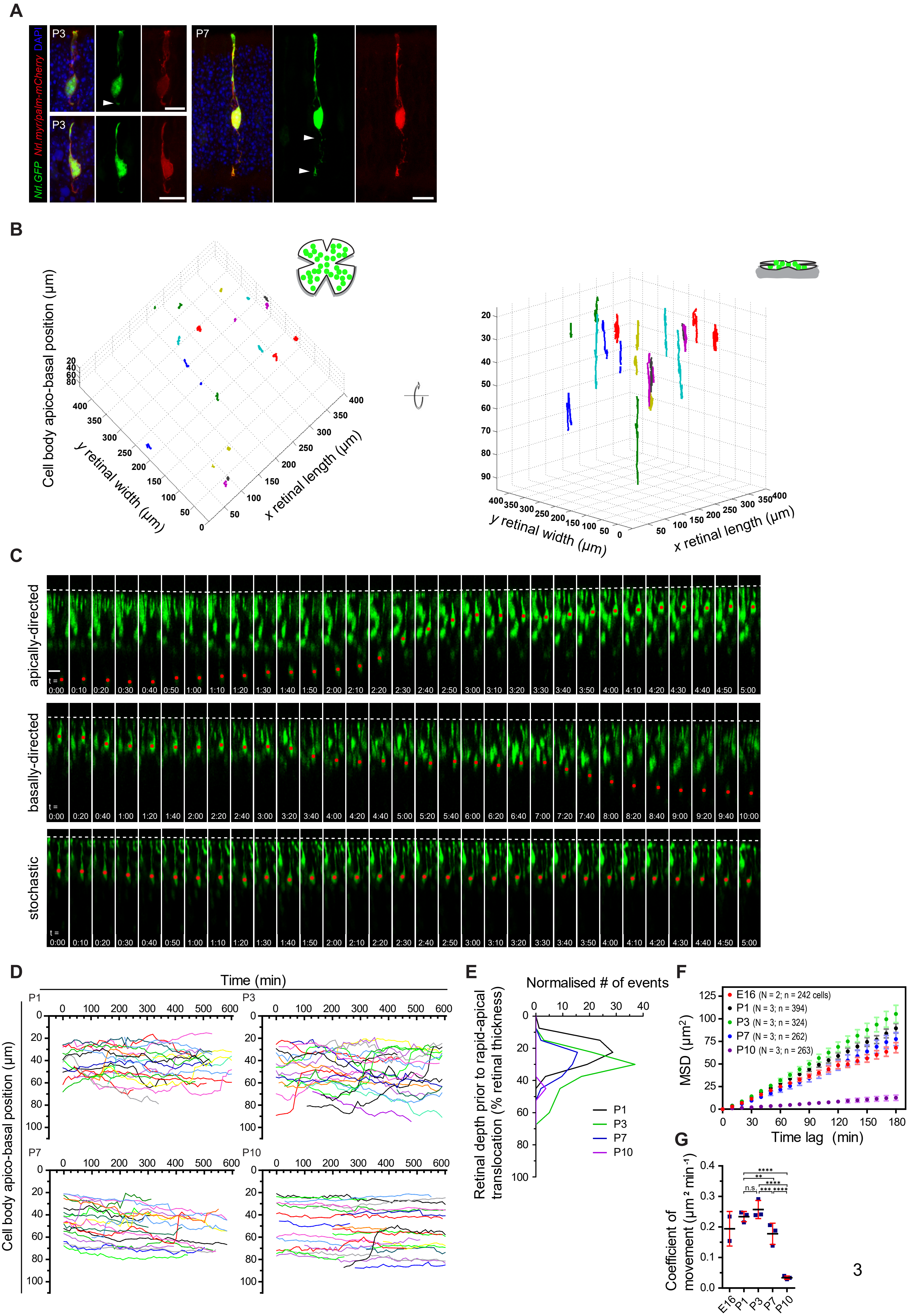

A
Nrl.GFP Nrl.myr/palm-mCherry DAPI
P3
P3
P7
B
Cell body apico-basal position (µm)
y retinal width (µm)
x retinal length (µm)
C
apically-directed
basally-directed
stochastic
t =
D
Time (min)
P1
P3
P7
P10
Cell body apico-basal position (µm)
E
Normalised # of events
Retinal depth prior to rapid-apical translocation (% retinal thickness)
P1
P3
P7
P10
F
E16 (N = 2; n = 242 cells)
P1 (N = 3; n = 394)
P3 (N = 3; n = 324)
P7 (N = 3; n = 262)
P10 (N = 3; n = 263)
MSD (µm²)
Time lag (min)
G
Coefficient of movement (µm² min⁻¹)
n.s.
E16
P1
P3
P7
P10

3

Supplementary Fig. S1. Rod photoreceptor nuclei undergo apico-basal translocations during retinogenesis.

Related to Fig. 1.

(A) Expression of cytoplasmic *Nrl.EGFP* (green) and membrane-targeting *Nrl.myr/palm-mCherry* (red) in wild-type retinae counterstained with DAPI (blue) to visualise the basal process (arrowhead). Electroporation was performed at P1. At P3, the basal process was not always present and could be of variable length. Where a basal process was detected, membrane label (*myr/palm-mCherry*) was frequently evident in absence of cytoplasmic EGFP (compare top vs bottom panels). At P7, both cytoplasmic and membrane label were visible in most cases (right panels). **(B)** Live-imaged field of view (*xyzt*) of a flatmounted P3 *Nrl.GFP*$^{+/+}$ retina showing the same representative nuclear trajectories as viewed from different angles. **(C)** Time lapse recording of a manually segmented rod (green) nucleus (red dot) migrating along the apico-basal cellular axis in a P3 *Nrl.GFP*$^{+/+}$ retina. Apical (top) and basal (middle) movements, as well as non-directional periods with no net positional change (bottom series) are shown. See also Supplementary movies S2-4. Dotted line indicates apical retinal limit. **(D)** Representative rod PR apico-basal nuclear trajectories observed in P1, P3, P7, and P10 retinae superimposed in a single *zt* plot. Each individual trajectory represents an individual nuclear trajectory. **(E)** Distribution of apico-basal starting positions of rapid apical nuclear translocations at P1-P10 expressed as % of retinal thickness. Retinal thickness measurements were obtained from fixed reference retinae at each of the time points. **(F)** Mean squared displacement (MSD) profiles of pooled rod nuclear translocations observed from E16-P10. **(G)** Coefficients of movement (directly proportional to the slopes of the MSD curves; see methods) are shown for each time point from E16-P10. Experimental repeats, each containing a whole set of nuclear trajectories, were collapsed down to individual data points (blue). Time point E16 only consists of 2 experimental repeats and was thus not included in the statistical analysis. Scale bars, 10 µm. 2-way ANOVA with post-hoc permutation test. n.s., not significant; *$p < 0.05$; **$p < 0.01$; ***$p < 0.001$; ****$p < 0.0001$. Data show mean ± SEM (**F**).

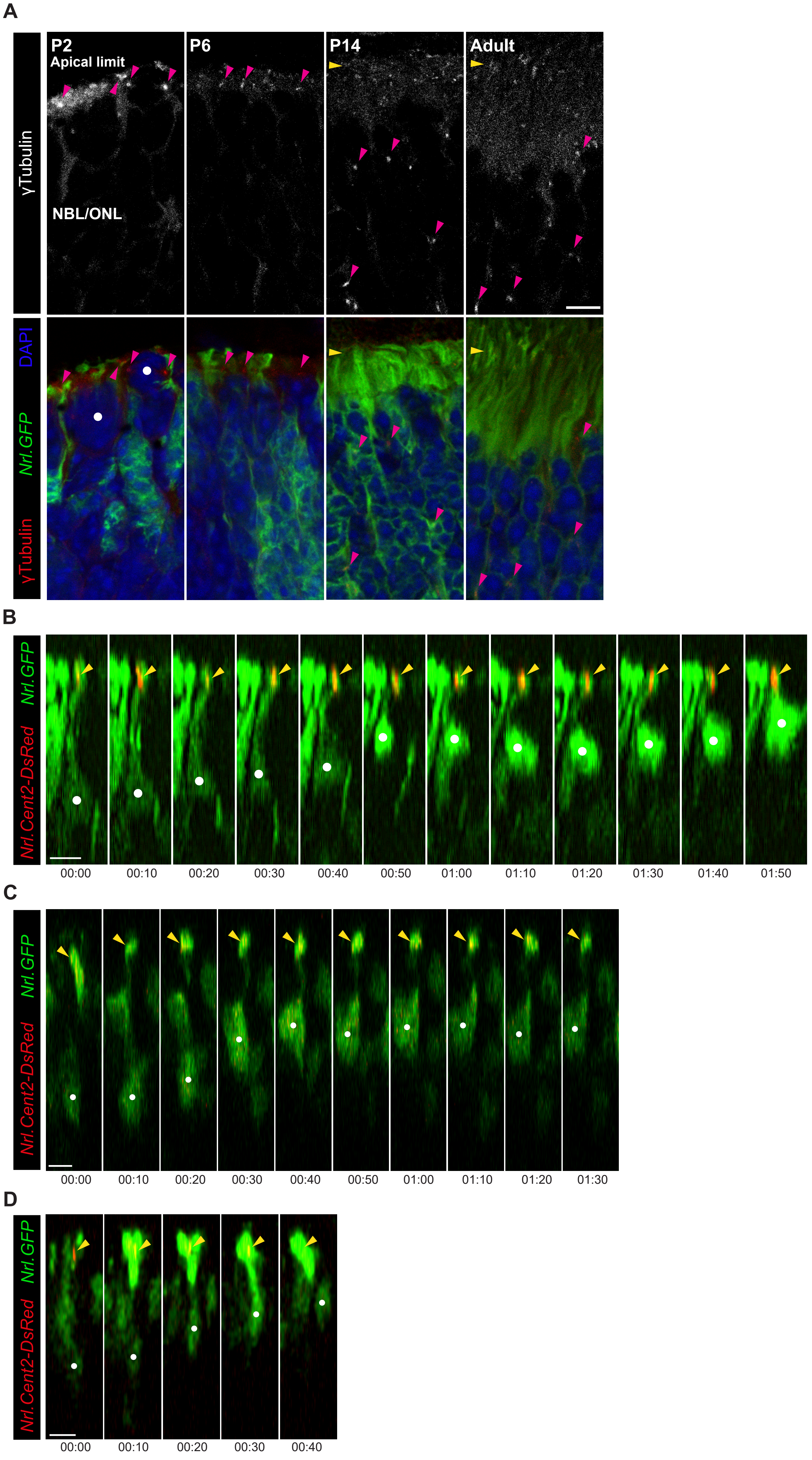
A
P2
Apical limit
NBL/ONL
P6
P14
Adult
γTubulin
DAPI
Nrl.GFP
γTubulin
B
Nrl.GFP
Nrl.Cent2-DsRed
00:00
00:10
00:20
00:30
00:40
00:50
01:00
01:10
01:20
01:30
01:40
01:50
C
Nrl.GFP
Nrl.Cent2-DsRed
00:00
00:10
00:20
00:30
00:40
00:50
01:00
01:10
01:20
01:30
D
Nrl.GFP
Nrl.Cent2-DsRed
00:00
00:10
00:20
00:30
00:40

Supplementary Fig. S2. Rod nuclear motility does not require motile centrosomes.

Related to Fig. 2.

(A) γ-tubulin (grey in top panel, red in lower panel) labels all centrosomes. At P2, labelling is restricted to the apical margin but becomes more dispersed by P8 as post-mitotic neurons migrate away. **(B-D)** Time lapse series of explanted P1 *Nrl.GFP*$^{+/+}$ retinae (green) electroporated with *Nrl.Cent2-DsRed* (red) and cultured *in vitro* for 3 days. The centrosomes remain at the apical tissue limit (yellow arrowhead) during rapid apically-directed nuclear translocation (white dot). Scale bars, 5 µm.

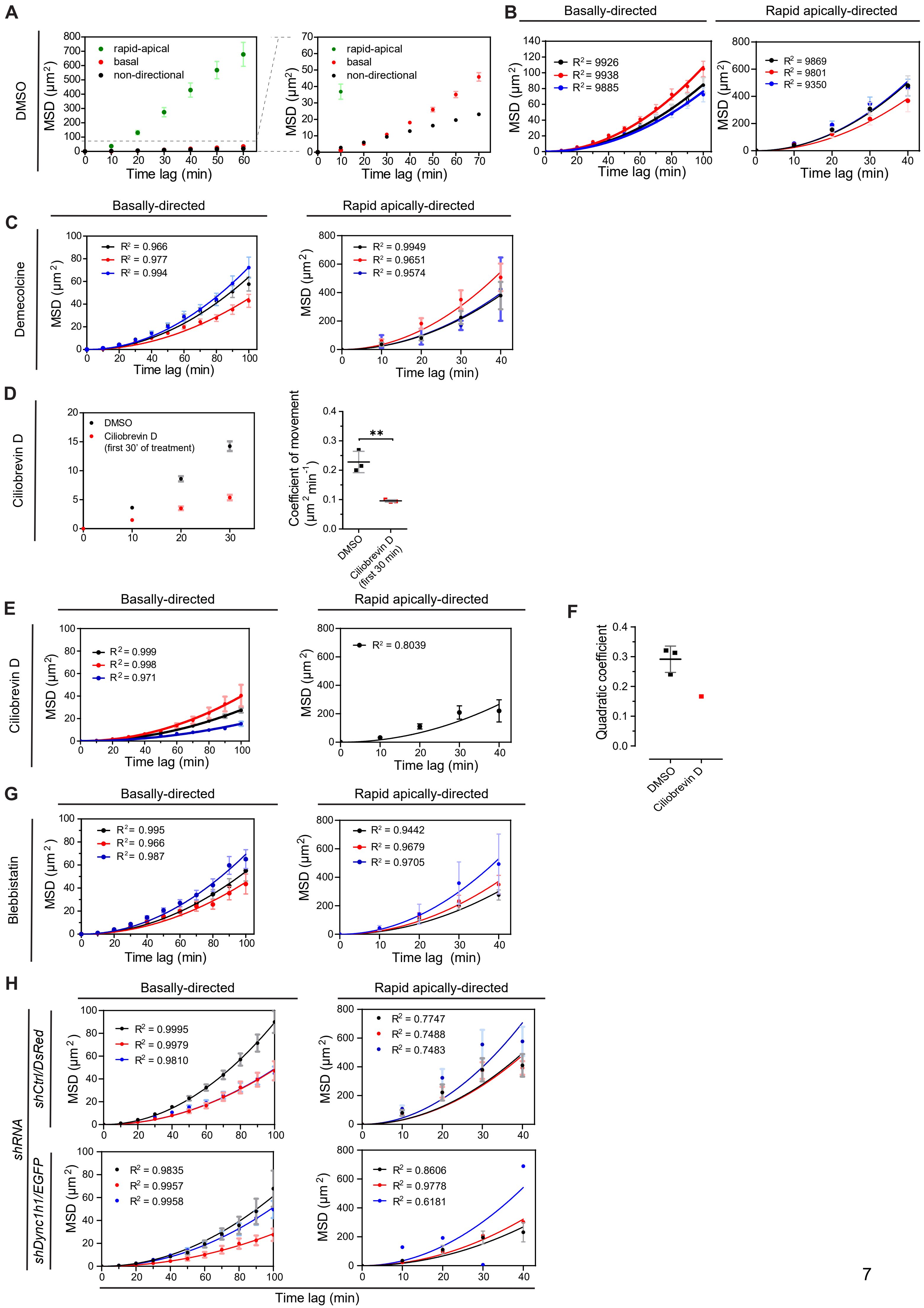

A
DMSO
rapid-apical
basal
non-directional
MSD (μm²)
Time lag (min)
B
Basally-directed
Rapid apically-directed
R² = 9926
R² = 9938
R² = 9885
R² = 9869
R² = 9801
R² = 9350
C
Demecolcine
R² = 0.966
R² = 0.977
R² = 0.994
R² = 0.9949
R² = 0.9651
R² = 0.9574
D
Ciliobrevin D
DMSO
Ciliobrevin D
(first 30' of treatment)
Coefficient of movement (μm² min⁻¹)
**
Ciliobrevin D (first 30 min)
E
R² = 0.999
R² = 0.998
R² = 0.971
R² = 0.8039
F
Quadratic coefficient
G
Blebbistatin
R² = 0.995
R² = 0.966
R² = 0.987
R² = 0.9442
R² = 0.9679
R² = 0.9705
H
shRNA
shCtrl/DsRed
shDync1h1/EGFP
R² = 0.9995
R² = 0.9979
R² = 0.9810
R² = 0.7747
R² = 0.7488
R² = 0.7483
R² = 0.9835
R² = 0.9957
R² = 0.9958
R² = 0.8606
R² = 0.9778
R² = 0.6181

Supplementary Fig. S3. Mean squared displacement (MSD) comparison of rapid apical, basal and non-directional rod nuclear migration.

Related to Fig. 1-5.

(A) MSD profiles of total rapid apical, basal and non-directional rod nuclear translocations in P3 retinae. Right panel displays magnified portion of left panel. Data show mean ± SEM. **(B, C, E, G)** MSD profiles of *total* basal (left panels) and *rapid-apical* rod nuclear translocations (right panels) in P3 retinae exposed to DMSO control **(B)**, Demecolcine **(C)**, Ciliobrevin D **(E)**, and Blebbistatin **(G)**. Different MSD profiles (black, red and blue) are associated with the 3 performed independent experimental repeats in each condition. **(D)** MSD profiles of *total* rod nuclear translocations for initial 30 min of Ciliobrevin D treatment compared with DMSO; corresponding coefficients of movement are shown on the right. In (E), a small number of rapid apical nuclear translocations was observed in 1 of the 3 experimental repeats, thus producing only 1 instead of 3 MSD profiles. **(H)** MSD profiles of *total* basal (left panels) and *rapid-apical* rod nuclear translocations (right panels) in *Nrl.Cre*$^{+/-}$ retinae following electroporation with *shCtrl/DsRed* or *shDync1h1/EGFP* at P1 and culturing for 6DIV. MSD profiles for rapid apically- and basally-directed nuclear translocations throughout this figure were curve fitted with the quadratic function $(y = ax^2)$. Data show mean ± SEM for MSD data points, mean ± SD for coefficient of movement and quadratic coefficient plots. Goodness-of-fit was assessed by R^2 analysis and results are shown in each plot; unpaired *t* test; $^{**}p < 0.01$.

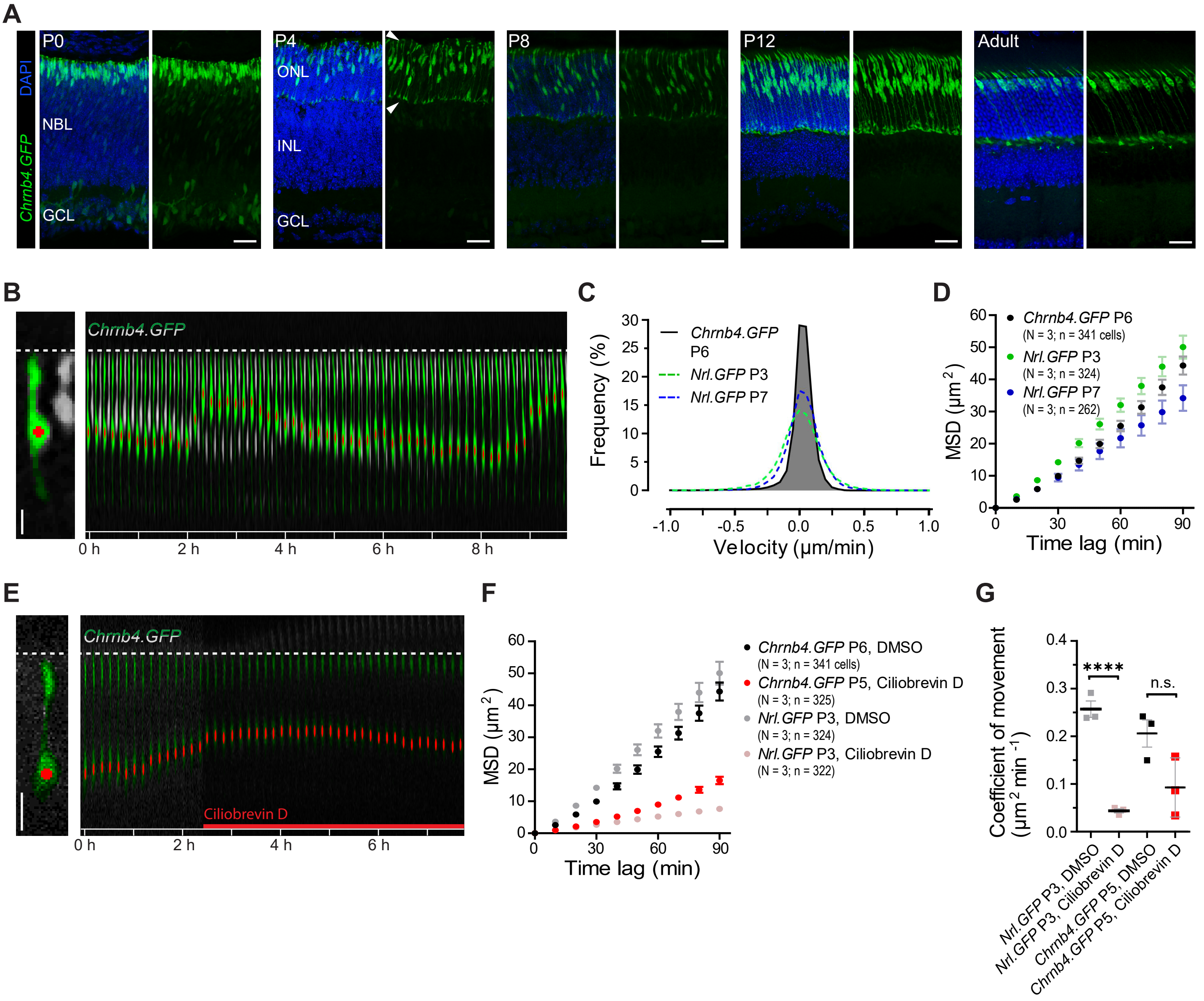
A
DAPI
Chrnb4.GFP
P0
NBL
GCL
P4
ONL
INL
GCL
P8
P12
Adult
B
Chrnb4.GFP
0 h
2 h
4 h
6 h
8 h
C
Frequency (%)
Chrnb4.GFP P6
Nrl.GFP P3
Nrl.GFP P7
Velocity (µm/min)
D
MSD (µm²)
Chrnb4.GFP P6 (N = 3; n = 341 cells)
Nrl.GFP P3 (N = 3; n = 324)
Nrl.GFP P7 (N = 3; n = 262)
Time lag (min)
E
Chrnb4.GFP
Ciliobrevin D
0 h
2 h
4 h
6 h
F
MSD (µm²)
Chrnb4.GFP P6, DMSO (N = 3; n = 341 cells)
Chrnb4.GFP P5, Ciliobrevin D (N = 3; n = 325)
Nrl.GFP P3, DMSO (N = 3; n = 324)
Nrl.GFP P3, Ciliobrevin D (N = 3; n = 322)
Time lag (min)
G
Coefficient of movement (µm² min⁻¹)
****
n.s.
Nrl.GFP P3, DMSO
Nrl.GFP P3, Ciliobrevin D
Chrnb4.GFP P5, DMSO
Chrnb4.GFP P5, Ciliobrevin D

Supplementary Fig. S4. Cone photoreceptors undergo dynein-dependent apico-basal nuclear translocations.

Related to Figs. 1, 4.

(A) *EGFP* expression in the *Chrnb4-EGFP* retina at various developmental stages and in the adult mouse. White arrow heads at P4 indicate apical and basal cone processes. **(B)** Time lapse series of a manually segmented cone (green) nucleus (red dot) migrating in the apico-basal cellular axis in P3 *Chrnb4.EGFP* retina (grayscale). **(C)** Comparison of total cone nuclear velocity distributions at P6 (*Chrnb4.EGFP*) with total rod nuclear velocity distributions at P3 and P7 (*Nrl.GFP*$^{+/+}$). **(D)** MSD profiles of total cone nuclear translocations observed at P6 (*Chrnb4.EGFP*) compared with total rod nuclear velocity distributions at P3 and P7 (*Nrl.GFP*$^{+/+}$). **(E)** Time lapse of a manually segmented cone (green) nucleus (red dot) migrating in the apico-basal cellular axis in a P5 *Chrnb4.EGFP* retina (grayscale) exposed to Ciliobrevin D from 120 min onwards. **(F)** MSD profiles of total cone nuclear translocations in Ciliobrevin D vs DMSO, compared with MSD profiles of total rod nuclear translocations at P3. **(G)** Coefficients of movement for total cone nuclear translocations in Ciliobrevin D vs DMSO and total rod nuclear translocations in P3 retinae. Experimental repeats, each containing a whole set of nuclear trajectories, were collapsed down to individual data points. Scale bars, (**A**) 25 µm, (**B, E**) 5 µm. Unpaired t test; n.s., not significant; ****$p < 0.0001$. Data show mean ± SEM (**D, F**).

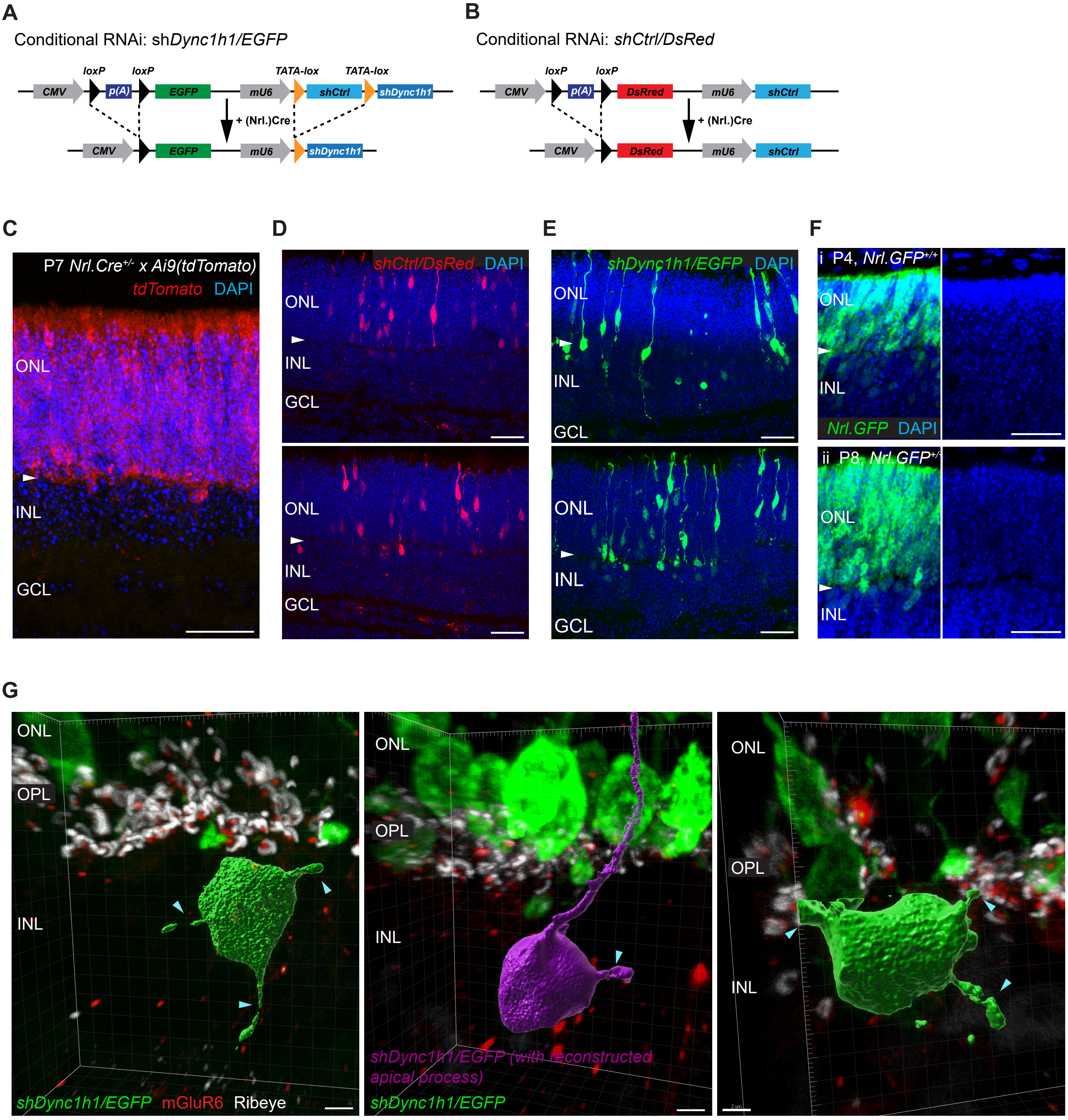
A
Conditional RNAi: shDync1h1/EGFP
loxP
loxP
TATA-lox
TATA-lox
CMV
p(A)
EGFP
mU6
shCtrl
shDync1h1
+ (Nrl.)Cre
CMV
EGFP
mU6
shDync1h1
B
Conditional RNAi: shCtrl/DsRed
loxP
loxP
CMV
p(A)
DsRred
mU6
shCtrl
+ (Nrl.)Cre
CMV
DsRed
mU6
shCtrl
C
P7 Nrl.Cre+/- x Ai9(tdTomato)
tdTomato DAPI
ONL
INL
GCL
D
shCtrl/DsRed DAPI
ONL
INL
GCL
ONL
INL
GCL
E
shDync1h1/EGFP DAPI
ONL
INL
GCL
ONL
INL
GCL
F
i P4, Nrl.GFP+/+
ONL
INL
Nrl.GFP DAPI
ii P8, Nrl.GFP+/-
ONL
INL
G
ONL
OPL
INL
shDync1h1/EGFP mGluR6 Ribeye
ONL
OPL
INL
shDync1h1/EGFP (with reconstructed apical process)
shDync1h1/EGFP
ONL
OPL
INL

Supplementary Fig. S5. Conditional dynein 1 loss-of-function in rods results in ectopically located photoreceptors and disrupted ONL lamination.

Related to Figs. 5, 6.

(A) Schematic of *shDync1h1/EGFP* gene silencing construct for conditional Cre-mediated (*Nrl.Cre*$^{+/-}$ mouse) short hairpin (*shDync1h1*) and reporter gene (EGFP) expression in rods. **(B)** Schematic of control construct for conditional Cre-mediated scrambled *shCtrl* short hairpin and *DsRed* expression. **(C)** Cre expression is restricted to rod PRs in P4 *Nrl.Cre*$^{+/-}$ *x Ai9* mice, as previously reported by Brightman et al, 2016 ([55]). The *Ai9* mouse strain is a ubiquitous Cre reporter line, producing tdTomato expression in cells where Cre is expressed.[75] Note that tdTomato-expressing rod cells can be observed in the ONL as well as in ectopic locations (OPL, INL). **(D, E)** Apico-basal positions of transfected rods expressing *shCtrl/DsRed* (**(D)**; red) or *shDync1h1/EGFP* (**(E)**; green) in *Nrl.Cre*$^{+/-}$ retina following electroporation at P1 and culturing for 4DIV. Arrowhead indicates the OPL. Note that basally displaced *shCtrl/DsRed* expressing rods were also be observed (**(D)**, bottom panel), albeit at lower numbers compared with *shDync1h1/EGFP* expressing rods **(E)**. **(F)** Rod PR somata in ectopically basal locations beyond the OPL in the P4 (i) and P8 (ii) *Nrl.GFP*$^{+/+}$ retina. Arrowhead indicates the OPL. **(G)** 3D representations of basally-displaced rod PRs virally transduced with *AAV2/8 shDync1h1/EGFP* (green) at P1 and harvested 3 wks post viral administration. Retinae were immunolabelled for ribeye (grayscale) and mGluR6 (red). Basally-displaced cells frequently exhibit lateral and/or basal processes (cyan arrowheads). Middle panel shows 3D rendered basally displaced shDync1h1/EGFP^{+ve} rod with traceable apical process (magenta). Scale bars, (**C-F**) 25 µm; **(G)** 3 µm.
