# Supplementary material for: Repeated nuclear translocations underlie photoreceptor positioning and lamination of the outer nuclear layer in the mammalian retina
Source: Cell Rep. 2021 Aug 3;36(5):109461. doi: 10.1016/j.celrep.2021.109461 (PMC8356022; doi:10.1016/j.celrep.2021.109461)
Supplement: Document S2. Article plus supplemental information [file mmc15.pdf]

Cell Reports

Article

Repeated nuclear translocations underlie photoreceptor positioning and lamination of the outer nuclear layer in the mammalian retina

Graphical abstract

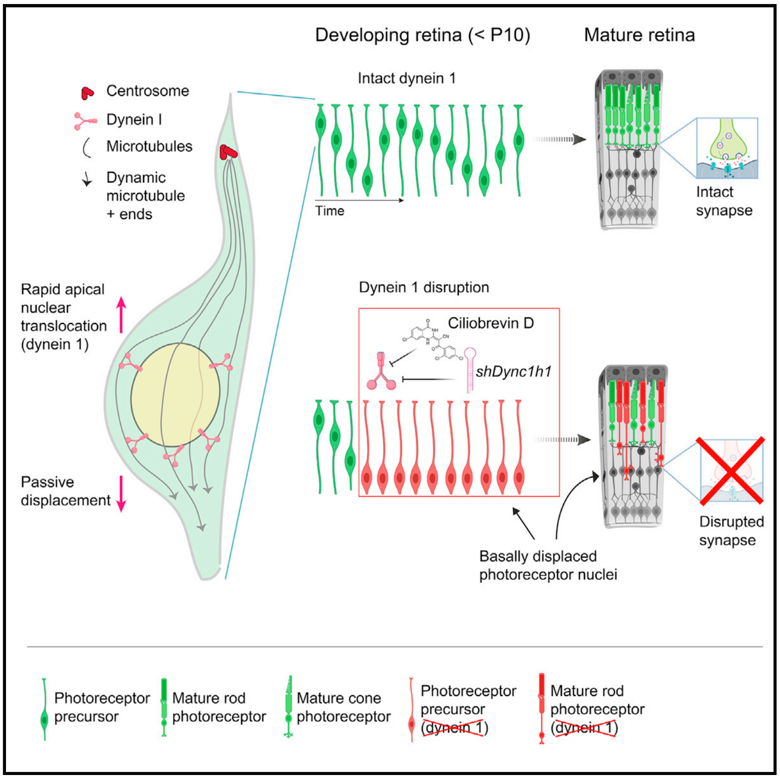

Highlights

- Photoreceptor nuclei exhibit apico-basal motility in the developing mouse retina
- Rapid apically directed nuclear translocation is powered by dynein 1
- Dynein 1 disruption impedes rapid apical nuclear translocation, causing displacement
- Synapse formation is disrupted in basally displaced photoreceptors

Authors

Nozie D. Aghaizu, Katherine M. Warre-Cornish, Martha R. Robinson, ..., Alexander J. Smith, Robin R. Ali, Rachael A. Pearson

Correspondence

nozie.aghaizu@ucl.ac.uk (N.D.A.), rachael.pearson@kcl.ac.uk (R.A.P.)

In brief

Photoreceptors occupy a defined layer in the retina. Aghaizu et al. show that mammalian rod and cone photoreceptors use repeated, dynein-1-driven apically directed nuclear translocations for fine-positioning and retention within this layer. Dynein 1 disruption in photoreceptors results in ectopic displacement beyond the photoreceptor layer and impaired synapse formation.

Aghaizu et al., 2021, Cell Reports *36*, 109461
August 3, 2021

© 2021 The Authors.

https://doi.org/10.1016/j.celrep.2021.109461

CellPress

Cell Reports

CellPress
OPEN ACCESS

Article

Repeated nuclear translocations underlie photoreceptor positioning and lamination of the outer nuclear layer in the mammalian retina

Nozie D. Aghaizu,[1,3,*] Katherine M. Warre-Cornish,[1] Martha R. Robinson,[1] Paul V. Waldron,[1] Ryea N. Maswood,[1] Alexander J. Smith,[1,2] Robin R. Ali,[1,2] and Rachael A. Pearson[1,2,4,5,*]
[1]University College London Institute of Ophthalmology, London EC1V 9EL, UK
[2]Centre for Cell and Gene Therapy, King's College London, Guy's Hospital, London SE1 9RT, UK
[3]Present address: Dementia Research Institute, University College London, London WC1E 6BT, UK
[4]Lead contact
[5]Senior author
*Correspondence: nozie.aghaizu@ucl.ac.uk (N.D.A.), rachael.pearson@kcl.ac.uk (R.A.P.)
https://doi.org/10.1016/j.celrep.2021.109461

SUMMARY

In development, almost all stratified neurons must migrate from their birthplace to the appropriate neural layer. Photoreceptors reside in the most apical layer of the retina, near their place of birth. Whether photoreceptors require migratory events for fine-positioning and/or retention within this layer is not well understood. Here, we show that photoreceptor nuclei of the developing mouse retina cyclically exhibit rapid, dynein-1-dependent translocation toward the apical surface, before moving more slowly in the basal direction, likely due to passive displacement by neighboring retinal nuclei. Attenuating dynein 1 function in rod photoreceptors results in their ectopic basal displacement into the outer plexiform layer and inner nuclear layer. Synapse formation is also compromised in these displaced cells. We propose that repeated, apically directed nuclear translocation events are necessary to ensure retention of post-mitotic photoreceptors within the emerging outer nuclear layer during retinogenesis, which is critical for correct neuronal lamination.

INTRODUCTION

The central nervous system is characterized by its stratified organization, and the arrangement of newly born neurons into distinct layers is critical for synaptic connectivity and function. In the vertebrate retina, photoreceptors (PRs) reside exclusively in the outer nuclear layer (ONL) (Figure 1A). They are bordered apically by the retinal pigment epithelium (RPE), which supports PR function and survival. Basal to the ONL lies the interneurons of the inner nuclear layer (INL), which form synaptic connections to PRs within the outer plexiform layer (OPL). The accurate positioning of PRs between the RPE and INL facilitates the establishment of correctly located PR synapses, which is essential for vision (Dick et al., 2003; Maddox et al., 2015; Sarin et al., 2018).

In development, retinal progenitor cells (RPCs) undergo mitosis at the apical limit of the retina (Figure 1A). Consequently, most post-mitotic daughter cells must move basally into their designated strata and do so by using a variety of methods; retinal ganglion cells (RGCs) migrate across the entire retinal radial width by using fast, unidirectional somal translocation (Icha et al., 2016; Poggi et al., 2005; Zolessi et al., 2006), whereas horizontal cells initially overshoot the INL during bipolar migration, before returning apically by multipolar migration (Chow et al., 2015; Edqvist and Hallböök, 2004). The nascent ONL overlaps with the neuroblastic layer (NBL) that exists before the ONL and INL separate with the forming OPL (Sarin et al., 2018). Cone PRs are born early in development and are initially displaced throughout the NBL (Suzuki et al., 2013), before becoming restricted to their adult location at the apical margin of the ONL (Rich, et al., 1997).

Cone PR nuclei achieve this apical position by a nuclear translocation mechanism that involves Linker of Nucleoskeleton and Cytoskeleton (LINC). LINC complexes are involved in centrosome-mediated nuclear translocations, for which mechanical forces are exerted by kinesin-1 and dynein, which interact with KASH (Klarsicht, ANC-1, Syne homology) proteins (Fridolfsson et al., 2010; Fridolfsson and Starr, 2010; Zhang et al., 2009). Disruption of LINC complexes resulted in partially mis-localized PR nuclei in the adult mouse (Razafsky et al., 2012; Yu et al., 2011), whereas in zebrafish, exogenous expression of the KASH domain of Syne2a induced PR nuclei mispositioning (Tsujikawa et al., 2007). Similar phenotypes are reported in *Drosophila klarsicht* (Nesprin) (Patterson et al., 2004), *klaroid* (Sun) (Kracklauer et al., 2007), and *glued* (dynactin) (Whited et al., 2004) and in zebrafish mok (dynactin) mutants (Tsujikawa et al., 2007).

Unlike cones, rod somata are distributed throughout the ONL, but there is a gap in our understanding regarding potential migratory phenomena required for rod somal positioning and/or retention within the ONL. Histological studies in mice (Akimoto et al., 2006; Sarin et al., 2018; Young, 1984) and in

This is an open access article under the CC BY license (http://creativecommons.org/licenses/by/4.0/).

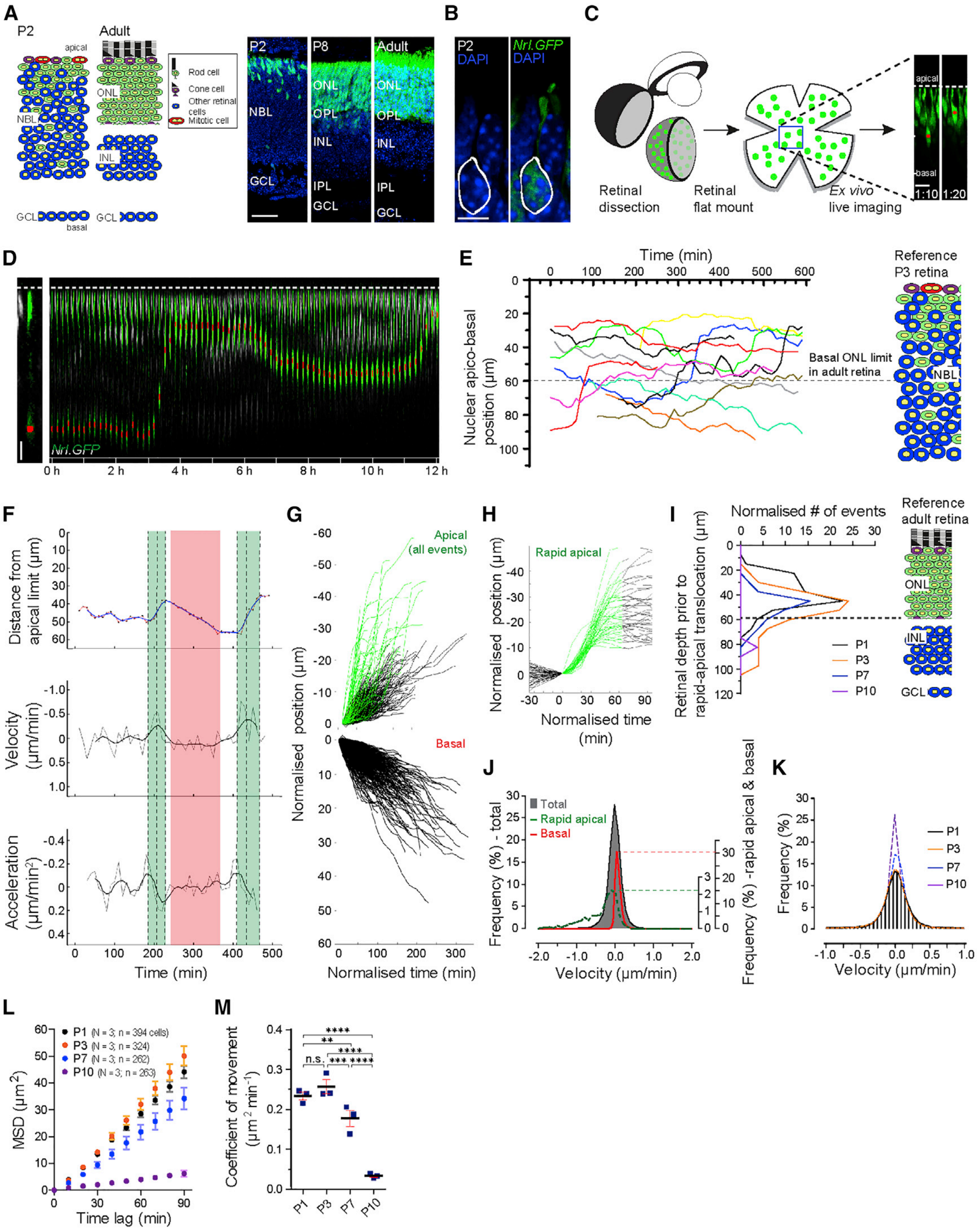

Figure 1. Rod photoreceptor nuclei are motile during retinogenesis

(A) Location of rods (green) in the developing and adult *Nrl.GFP*$^{+/+}$ retina.

(B) Individual rod in P2 *Nrl.GFP*$^{+/+}$ retina. Soma is highlighted (white).

(C) Schematic of retinal preparation for time-lapse live imaging to track rod nuclear motions (red dots) in *Nrl.GFP* $^{+/+}$ retinae.

(D) Nucleus (red dot) of a segmented rod (green) migrating apico-basally in P3 *Nrl.GFP*$^{+/+}$ retina (grayscale).

(E) Overlaid example trajectories depicting apico-basal rod nuclear motility at P3. Basal ONL limit indicated (Ferguson et al., 2013).

(F) Representative kinetics of apico-basal rod nuclear motility at P3. Nuclear position, velocity, and acceleration plotted against time. Real data points (position: black dots/red line; velocity and acceleration: gray lines) and moving average (position: blue; velocity and acceleration: black) are shown. Periods of apical- and basal-directed movement are shaded green and red, respectively. Local velocity minima indicate peak of rapid apically directed translocation phase (middle dotted line). Local acceleration minima/maxima indicate initiation and cessation of rapid apically directed translocation (outer dotted lines).

(legend continued on next page)

human-stem-cell-derived retinal organoids (Kaewkhaw et al., 2015) indicate that early in development many newborn rod nuclei are basal to the limits of the eventual ONL. Similarly, in zebrafish, rod and cone PR somata can be found basal to the nascent ONL early in development (Suzuki et al., 2013). Because neither are found outside the ONL in the adult retina, it has been hypothesized that these displaced nuclei must move apically or back into the ONL or that the cells die (Young, 1984). We therefore sought to determine whether displaced PRs undergo a form of nuclear translocation and how this contributes to stratification of the mammalian ONL.

Here, we report that rod and cone PR nuclei undergo dynein-dependent apically directed nuclear translocation. More importantly, this is not a single positioning event, as perhaps envisaged by earlier descriptions of cone stratification. Instead, PR nuclei undergo repeated apical translocations as part of an oscillatory apico-basal cycle throughout early retinogenesis. Dynein 1 disruption impedes apical translocation and leads to lamina defects, with PRs being displaced into the OPL and INL and displaying compromised synapse formation. We propose that repeated, apical nuclear translocation events represent a pattern of movement not previously described for a post-mitotic neuron. In the retina, this serves to retain PRs within the forming ONL during retinogenesis and ensures correct neural stratification.

RESULTS

Rod PR nuclei are motile during retinogenesis

To study rod PR motility during mouse development, we examined *Nrl.Gfp*$^{+/+}$ mice from embryonic day 16 (E16) to postnatal day 10 (P10). In the retina, these mice express GFP specifically in post-mitotic rods under control of the *Nrl* promoter (Akimoto et al., 2006; Kim et al., 2016; Figure 1A). At this stage, rod PRs exhibit spindle-shaped somata (Figure 1B) and the nucleus is the principal occupant of the rod soma by volume (Figure 1B). Thus, somata position may be taken as the position of the nucleus. Imaging of retinal explants revealed that rod somata were permanently anchored to the limit of the retina by an apical process. Conversely, basal processes were frequently too thin and/or weakly labeled to be detectable based on cytoplasmic GFP; instead, we used membrane labeling (*Nrl.myr/palm-mCherry*) for visualization (Figure S1A), which showed them to be ubiquitous from P7, a time that coincides with the ramification of the OPL (Huckfeldt et al., 2009; Morgan et al., 2006).

Real-time imaging of explanted retinae revealed that between P1–P7 most rod nuclei are highly motile, moving in the apico-basal (radial) axis, with little or no lateral movement (Figure 1D; Figure S1B; Video S1). At P1, we detected 493 basally and 307 apically directed movements (classified by exhibiting persistent positive and negative velocities, respectively, for at least 1 h) within a total population of 394 rod nuclear trajectories (N = 3 retinal explants). Each of them could be interspersed with periods of little/no net movement (Figures 1D and 1E; Figure S1C and Videos S2, S3, and S4 show representative examples of these movements at P3). Strikingly, a small proportion of rod nuclei could be observed undergoing more than one complete oscillation within the 12-h imaging period (~3%; n = 11/394 nuclei, N = 3 at P1; Figures 1D–1F; Video S5). This corresponds to 0.1 ± 0.0 oscillatory events per 1,000 recording mins (event count normalized by cumulative trajectory recording time per live imaged retina). In these presumptive repeated oscillations, the nuclei translocated rapidly in the apical direction, before moving more slowly in the basal direction, followed by another rapid apical translocation.

Within the population of net apically directed nuclear movements, we identified a distinct subgroup of high-speed (>−0.3 μm/min; range, −0.3 to −1.2) unidirectional translocations (Figure 1G; rapid apical phase, green), which when normalized for onset of movement, exhibited a highly uniform profile (Figure 1H). These events were non-synchronized and typically lasted ~1 h (Figures 1F and 1H). At P3, we observed 0.3 ± 0.1 rapid apical events per 1,000 recording mins (Table S1). Conversely, we found no evidence of an equivalent subgroup of comparably fast translocations in the basal direction (Figure 1G).

Here, the term rapid apical defines the group of fast, highly uniform, apically directed nuclear translocations. To differentiate between periods of little/no net movement and slow but persistent basal movements, we applied a threshold criterion for net basal movement of 15 μm (2 rod somal lengths) per 2 h. Where interventions were made, we compared the effects on total movement (rapid apical, basal, and non-directional), as well as the effects on rapid apical movements and basal movements, specifically.

We next addressed whether initiation of rapid apical translocation relates to a cell's depth within the retina (Figures 1I and S1E). Based on fixed sections, the P3 retina is ~160 μm thick. At this age, most rapid apical translocation events were initiated from 45–50 μm away from the apical margin, but a wide range

(G) Overlaid apically directed (top) and basally directed (bottom) events from a single recording, normalized with respect to the onset of movement at P3 (black/green traces). Green traces show rapid apical movements according to threshold criteria (see STAR Methods).

(H) Isolated, above-threshold rapid apical movements normalized as in (G).

(I) Distribution of apico-basal starting positions of rapid apical nuclear translocations at P1–P10 normalized with respect to cumulative recording time (sum of trajectory durations) for each condition.

(J) Velocity distribution of total (gray), rapid apical (green), and basal (red) rod nuclear movements at P3. The latter two are scaled up for clarity because component data points were only 2.2% and 30.1% of the number of total data points, respectively.

(K) Total rod nuclear velocity distributions at P1–P10.

(L) Mean squared displacement (MSD) profiles of total pooled rod nuclear translocations at P1–P10. Data show mean ± SEM. See also Figure S3A.

(M) Coefficients of movement at P1–P10. Individual data points (blue) represent experimental repeats, with each containing a whole set of nuclear trajectories. Two-way ANOVA with post hoc permutation test. Scale bars, 25 μm (A) and 5 μm (B and D). n.s., not significant; *$p < 0.05$; **$p < 0.01$; ***$p < 0.001$; ****$p < 0.0001$. Abbreviations: GCL, ganglion cell layer; INL, inner nuclear layer; IPL, inner plexiform layer; NBL, neuroblastic layer; ONL, outer nuclear layer; OPL, outer plexiform layer. See Figure S1 for more information.

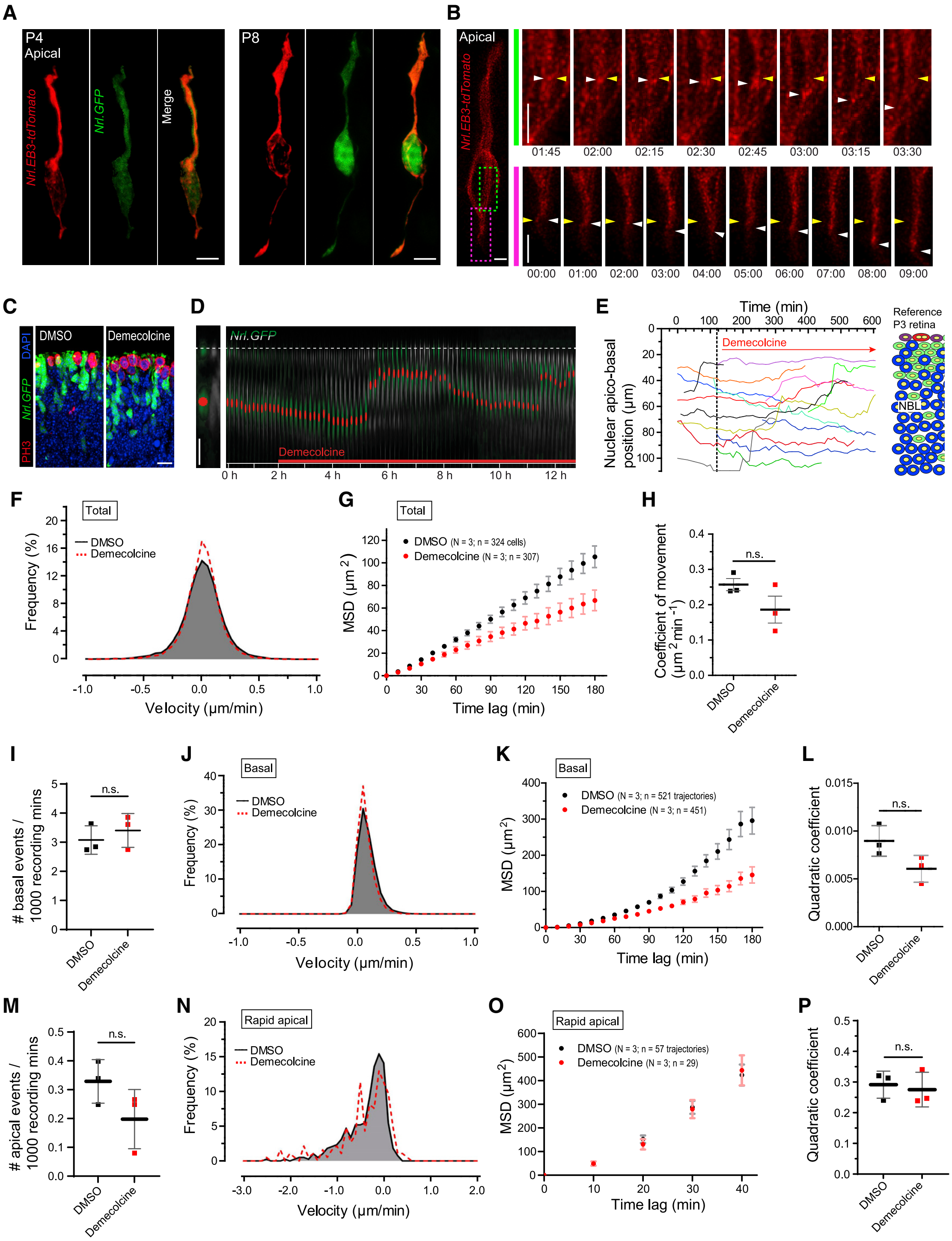

Figure 2. Rod nuclear motility does not require dynamic microtubule polymerization

(A) Rod MT plus ends labeled with *Nrl*.EB3-tdTomato (red) in segmented *Nrl.GFP*$^{+ve}$ rods (green) at P4 and P8.

(B) Time-lapse series of dynamic MT plus ends in rods labeled with *Nrl*.EB3-tdTomato (red) at 4 days post-retinal electroporation at P0. White arrowheads show moving EB3-tdTomato foci; yellow arrowheads demarcate starting position in the peri-nuclear fork (green region of interest [RO) and growing axon (magenta ROI).

(C) P3 *Nrl.GFP*$^{+/+}$ retina (green) following exposure for 10 h to 45 nM demecolcine leading to accumulation of PH3^{+ve} mitotic figures (red).

(D) Time-lapse series of a segmented rod (green) nucleus (red dot) moving apico-basally in a P3 *Nrl.GFP*$^{+/+}$ retina (grayscale) exposed to demecolcine, added at 120 min.

(legend continued on next page)

CellPress
OPEN ACCESS

(10–108 μm) was observed, with the deepest originating from a depth level with the future IPL (Ferguson et al., 2013). A similar distribution was observed at P1 and P7. Rapid apical translocations were virtually absent by P10; those that did occur were initiated from positions deep within the retina, basal to the nascent OPL. Thus, initiation of rapid apical translocations is not apparently tied to a specific apico-basal position within the tissue.

An analysis of all full-length rod nuclear trajectories at P3 revealed a quasi-Gaussian distribution of measured instantaneous velocities with a mean of 0.0 μm/min (Figure 1J). However, isolated rapid apical or basal nuclear movements exhibited instantaneous velocity profiles that were biased and skewed toward negative and positive velocity values, respectively.

Rod nuclear motility declines during development and becomes stationary by P10 (Figures 1K and 1L; Figure S1D; N = 3 experimental repeats (retinae) for each time point; P1: n = 394 individual nuclear trajectories, P3: n = 324, P7: n = 262, P10: n = 263; acquisition time = 10–12 h per retina; acquisition interval, Δt = 10 min). As noted above, apical and basal movements can be interspersed with considerable periods of little/no net movement. This manifests in a linear relationship between mean squared displacement (MSD) of rod nuclei with increasing time lag (Figures 1L and S1F for comparison with E16; see STAR Methods), a feature typical of particles undergoing non-directional motion (Ruthardt et al., 2011). The decline in overall nuclear motility was quantified by comparing the coefficient of movement, which is directly proportional to the slope of the MSD curves (see STAR Methods), and results are as follows: 0.234 ± 0.017 $\mu m^2\ min^{-1}$ at P1, 0.257 ± 0.030 $\mu m^2\ min^{-1}$ at P3, 0.178 ± 0.035 $\mu m^2\ min^{-1}$ at P7, and 0.034 ± 0.006 $\mu m^2\ min^{-1}$ at P10. Significant differences were found between ages (two-way ANOVA, $p = 0.029$), but not within groups ($p = 0.479$) (Figures 1M and S1G). The reduction in the coefficient of movement during development was highly significant according to post hoc permutation testing.

Taken together, these data suggest that post-mitotic rod PR nuclei undergo repeated rapid, apically directed translocation events, which are followed by periods of persistent basal drift that may be interspersed with periods of little or no net movement.

MT dynamic behavior is not required for rod nuclear oscillations

To investigate the molecular mechanisms underlying PR nuclear movements, we first examined the organization of the cytoskeleton (Kosodo, 2012). The microtubule (MT) cytoskeleton is comprised of MT filaments and MT organizing centers (MTOCs; centrosomes). In post-mitotic mammalian epithelial cells, the centrosome converts into the basal body (Hoyer-Fender, 2010). In developing rod PRs, the MTOC remains stationary at the top of the apical process at all times (Figures S2A–S2D). MT filaments contain a stable minus end located at the MTOC and a dynamic plus end that can extend or retract. To visualize MT plus-end dynamics, we expressed the plus-end binding protein EB3 fused to tdTomato (*Nrl.EB3-tdTomato*) (Merriam et al., 2013) in rods by *ex vivo* electroporation at P0–P1. TdTomato-labeled MTs were observed in transfected rods both at earlier (4 days *in vitro* [DIV]) and later stages (8 DIV) of retinogenesis (Figure 2A). Labeled MT bundles were seen in the apical process, wrapping around the nucleus (the perinuclear fork), and in the growing basal process (Figure 2A). MT plus ends (tdTomato^{+ve} foci) moved basally, indicating active MT polymerization (Figure 2B; Videos S6 and S7; Stepanova et al., 2003).

We next tested whether dynamic MT plus ends generate sufficient mechanical forces to translocate rod nuclei, as described for other cell types (Inoué and Salmon, 1995; Tran et al., 2001) by treating P3 retinal explants with demecolcine. MT-targeted drugs like demecolcine destabilize MTs at high doses (μM range). Indeed, 25 μM demecolcine led to tissue disintegration (data not shown). However, at low concentrations (nM range), demecolcine suppresses MT dynamics by attenuating MT plus-end (de-)polymerization without affecting cellular MT mass (Jordan and Wilson, 2004; Panda et al., 1995; Picone et al., 2010). Effective drug action at 45 nM was confirmed by the accumulation of PH3^{+ve} mitotic nuclei (which require dynamic MT plus-end polymerization for mitotic spindle formation) at the apical limit of the retina (Figure 2C) and by cessation of movement of EB3-tdTomato foci (Video S8).

Conversely, rod PR nuclear movement continued in the presence of 45 nM demecolcine (N = 3; n = 307; Figures 2D and 2E). The frequency distribution of total measured velocities was unaffected (Figure 2F), and although there was a small reduction in total movement, as shown by MSD and coefficient of movement analysis (Figures 2G and 2H), it was not statistically significant (DMSO: 0.257 ± 0.030 $\mu m^2\ min^{-1}$, demecolcine: 0.186 ± 0.066 $\mu m^2\ min^{-1}$; unpaired t test, $p = 0.165$). We next investigated whether demecolcine specifically affected either rapid apical or basal nuclear movements. There were no notable changes in event frequency and velocity profiles of rapid apical and basal movements in demecolcine- versus control-treated retinae (Figures 2I, 2J, 2M, and 2N; Table S1). Furthermore, we obtained MSD profiles for rapid apical and basal movements in both drug-treated and DMSO-control-treated retina that could be fitted with quadratic functions, yielding the associated quadratic coefficient (see STAR Methods; Figures S3B and S3C). There was a trend toward demecolcine reducing basal movement (Figures 2K, 2L and S3C; quadratic coefficients for DMSO: 0.009 ± 0.002, demecolcine: 0.006 ± 0.001; unpaired

(E) Representative, overlaid apico-basal rod nuclear trajectories at P3 in the presence of demecolcine after 120 mins.
(F–H) Effect of demecolcine versus DMSO on velocity distribution (F), MSD profiles (G), and coefficients of movement (H) for total rod nuclear movements. Data points represent experimental repeats; unpaired t test.
(I–L) Effect of demecolcine versus DMSO on frequency of basal events (I), velocity distribution (J), MSD profiles (K), and MSD profile-derived quadratic coefficients (L) for basal movements.
(M–P) Effect of demecolcine versus DMSO on frequency of apical events (M), velocity distribution (N), MSD profiles (O), and MSD profile-derived quadratic coefficients (P) for rapid apical nuclear translocations. Scale bars, 5 μm (A and B) and 10 μm (C and D). Unpaired t test; *$p < 0.05$; **$p < 0.01$. Data show mean ± SEM (G, K, and O). See also Figures S2B, S3B, and S3C.

t test, $p = 0.077$), but the MSD profile of rapid apical movements was unchanged (Figures 2O, 2P, S3B, and S3C; quadratic coefficients for DMSO: 0.292 ± 0.044, demecolcine: 0.275 ± 0.057; unpaired t test, $p = 0.717$). Although stable MTs likely play a role, these findings suggest that dynamic MT plus-end behavior is not critical for rapid apical movements of rod PR nuclei during development.

Rod nuclear oscillations do not require myosin II

The motor protein myosin II has been variously reported to mediate both apically directed (Norden et al., 2009) and basally directed (Schenk et al., 2009; Tsai et al., 2007) nuclear translocation in proliferating neuroepithelia, albeit in different animal and tissue models. Explanted P3 retinae were exposed to the myosin II selective antagonist blebbistatin (25 μM; dose selected based on published literature, including murine PRs) (Kovács et al., 2004; Norden et al., 2009; Reidel et al., 2008). Drug action was confirmed by an increase in $PH3^{+ve}$, M-phase arrested RPCs at the apical retinal limit (Figure 3A) that were unable to complete actomyosin-dependent cytokinesis (Straight et al., 2003). Conversely, time-lapse imaging revealed that drug-treated retinae (N = 3; n = 304) exhibited rod nuclear apico-basal motility similar to that of controls (Figures 3B–3D; compare Figure 1). The MSD profile for total movements was not significantly altered (Figure 3E) (coefficient of movement: 0.257 ± 0.030 for DMSO, 0.200 ± 0.068 μm^2 min^{-1} for blebbistatin; unpaired t test, $p = 0.259$) (Figure 3F).

The event frequency and velocity profiles of rapid apical and basal movements remained largely unchanged following drug administration (Figures 3G, 3H, and 3L; Table S1). There was a reduction in average displacement for basal movement (Figures 3I, 3J, S3B, and S3G; quadratic coefficient for DMSO: 0.009 ± 0.001, blebbistatin: 0.006 ± 0.001; unpaired t test, $p = 0.045$), whereas no reductions in average displacement for rapid apical nuclear translocations were observed (Figures 3M, 3N, S3B, and S3G; quadratic coefficient for DMSO: 0.292 ± 0.044, blebbistatin: 0.251 ± 0.073; unpaired t test, $p = 0.454$). Taken together, blocking myosin II constrictions mildly attenuates the kinetics of basally directed, but not rapid apical, nuclear movements, although the overall frequency of basal events was not affected.

Dynein 1 mediates rapid apical translocation of PR nuclei

As the MT polarity of rod PRs is similar to that of cortical progenitor cells (Kosodo et al., 2011; Troutt and Burnside, 1988; Tsai et al., 2010), which use dynein 1 to drive the nucleus apically during G2 of the cell cycle (Tsai et al., 2005, 2010), and LINC complexes appear to be involved in cone PR positioning (Razafsky et al., 2012; Tsujikawa et al., 2007), we considered dynein 1 to be a strong candidate to mediate repeated apically directed nuclear translocation in post-mitotic PRs.

We performed time-lapse live-imaging recordings on explanted P3 retinae treated with the dynein selective antagonist ciliobrevin D (25 μM; dose selected based on published literature) (Firestone et al., 2012; Herbert et al., 2017; Sainath and Gallo, 2014). Drug action was confirmed by basal mislocalization of the ciliary transport protein IFT88 (Figure 4A), which normally accumulates in the connecting cilium in a dynein-dependent manner (Sedmak and Wolfrum, 2011). In our live-imaging experiments, ciliobrevin D prevented most rapid apical nuclear movements within 20 mins of application (N = 3; n = 322; Figures 4B, 4C, and S3D; Table S1; Video S9). We also detected a robust concomitant attenuation of basal movements. Post-washout, rapid apical movements were restored (Figures 4D, 4F, and 4G; N = 2, n = 205). Note that prolonged exposure (>6 h) to ciliobrevin D led to tissue breakdown and cell death.

An analysis of total nuclear movements revealed a near-complete loss of higher-velocity measurements (Figure 4E). The slope of the MSD curve for ciliobrevin-D-treated retinae was reduced markedly relative to control (Figure 4F), together with a significant 81% reduction in the coefficient of movement (0.257 ± 0.030 versus 0.044 ± 0.007 μm^2 min^{-1}, respectively; unpaired t test, $p = 0.0001$; Figure 4G). Ciliobrevin D wash out restored movement to similar levels as DMSO control wash out (0.179 ± 0.015 versus 0.151 μm^2 min^{-1}, respectively; N = 1, n = 100). This finding indicates that short-term ciliobrevin D treatment is non-cytotoxic and fully reversible. That the coefficients of movement for ciliobrevin D and, especially, DMSO washout were below that of DMSO controls without washout most likely reflects the detrimental effect of prolonged tissue handling in the washout protocol.

Basal movements continued with a frequency similar to that seen in controls (Figure 4H; Table S1) but with lower velocities (Figure 4I) and shorter average displacement (Figures 4J, 4K, S3B, and S3E; quadratic coefficient for DMSO: 0.009 ± 0.002, ciliobrevin D: 0.003 ± 0.001; unpaired t test, $p = 0.006$). Rapid apical movements were almost completely abolished (n = 3 apical movements from N = 3 retinae; Figure 4L; Table S1). This makes the quantitative assessment of the kinetics of these few remaining events of limited value and accuracy (Figure 4M; note large error bars in Figure 4N). A quadratic coefficient analysis is provided in Figure S3F for completeness, but statistical comparisons were omitted.

Together, these initial pharmacological interventions support the hypothesis that dynein 1 is involved in rapid apical translocations of post-mitotic rod PR nuclei. Nota bene (N.B.) much higher doses of blebbistatin and demecolcine may also affect PR nuclear translocation, but given the significant effect of low doses of ciliobrevin, here, we focus on the role of dynein 1 in, and the biological purpose of, rapid apical translocation.

Cone PR precursors undergo apico-basal nuclear translocations that are kinetically and mechanistically similar to those of rods

As newly born mammalian cone PR nuclei are initially dispersed throughout the NBL (Figure S4A; Rich et al., 1997; Smiley et al., 2016; Waldron et al., 2018), we assessed whether cone nuclei also undergo repeated oscillatory movements, like rods. We performed real-time imaging of explanted retinae from *Chrnb4.EGFP* reporter mice (Gong et al., 2003), in which EGFP expression is predominantly restricted to cone PRs (Waldron et al., 2018). Cone nuclei exhibited apico-basal motility very similar to that of rods (Figure S4B; Video S13; N = 3, n = 341 cells). The velocity profile of total cone nuclear trajectories revealed a quasi-Gaussian distribution (mean, 0.0 μm/min), albeit with a notably increased low velocity contribution, compared

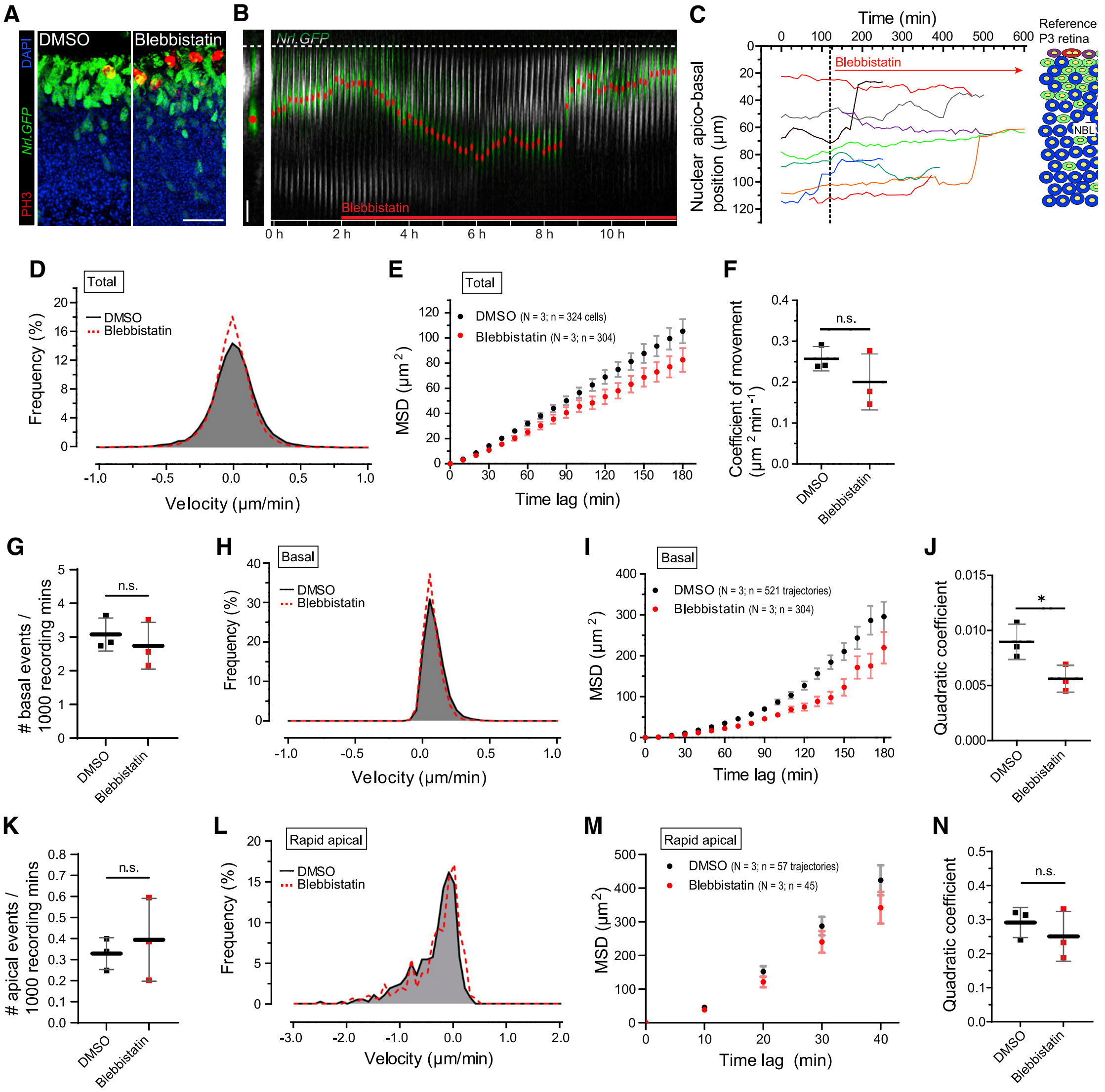

Figure 3. Rod nuclear translocation does not require actomyosin constrictions

(A) P3 *Nrl.GFP*$^{+/+}$ retina (green) following 10-h exposure to 25 µM blebbistatin resulting in accumulation of PH3^{+ve} mitotic figures (red).

(B) Time-lapse series of a segmented rod (green) nucleus (red dot) migrating apico-basally in P3 *Nrl.GFP*$^{+/+}$ retina (grayscale) exposed to blebbistatin, added after 120 min.

(C) Representative, overlaid apico-basal rod nuclear trajectories at P3 in the presence of blebbistatin after 120 mins.

(D–F) Effect of Blebbistatin versus DMSO on velocity distribution (D), MSD profiles (E), and coefficients of movement for MSD profiles (F) of total rod nuclear movements. Data points represent experimental repeats, unpaired t test.

(G–J) Effect of blebbistatin versus DMSO on frequency of basal events (G), velocity distribution (H), MSD profiles (I), and MSD-profile-derived quadratic coefficients (J) for basal movements.

(K–N) Effect of blebbistatin versus DMSO on frequency of apical events (K), velocity distribution (L), MSD profiles (M), and MSD-profile-derived quadratic coefficients (N) for rapid apical nuclear translocations. Scale bars, 10 µm (A) and 5 µm (B). Unpaired t test; $^{*}p < 0.05$; $^{**}p < 0.01$. Data show mean ± SEM (E, I, and M). See also Figures S3B and S3G.

with rod nuclei of a similar age (Figure S4C). Conversely, the average MSD was similar between cone and rod nuclei (Figure S4D). Next, we examined the effects of ciliobrevin D on cone nuclear motility (25 µM; N = 3; n = 325 cells); rapid apical translocations were virtually abolished upon drug exposure, whereas basal movements were attenuated, which is very similar to that seen for rods (compare Figure S4E with Figure 4). MSD analysis revealed a marked reduction in the total, average displacement (Figure S4F), similar in extent to that observed for rod nuclei, although this reduction was not statistically significant (DMSO: 0.206 ± 0.049 µm^2 min^{-1}, ciliobrevin D: 0.093 ± 0.062 µm^2 min^{-1}; unpaired t test, p = 0.068). Thus, repeated, dynein-dependent rapid apical movements are common to all post-mitotic PRs during retinogenesis.

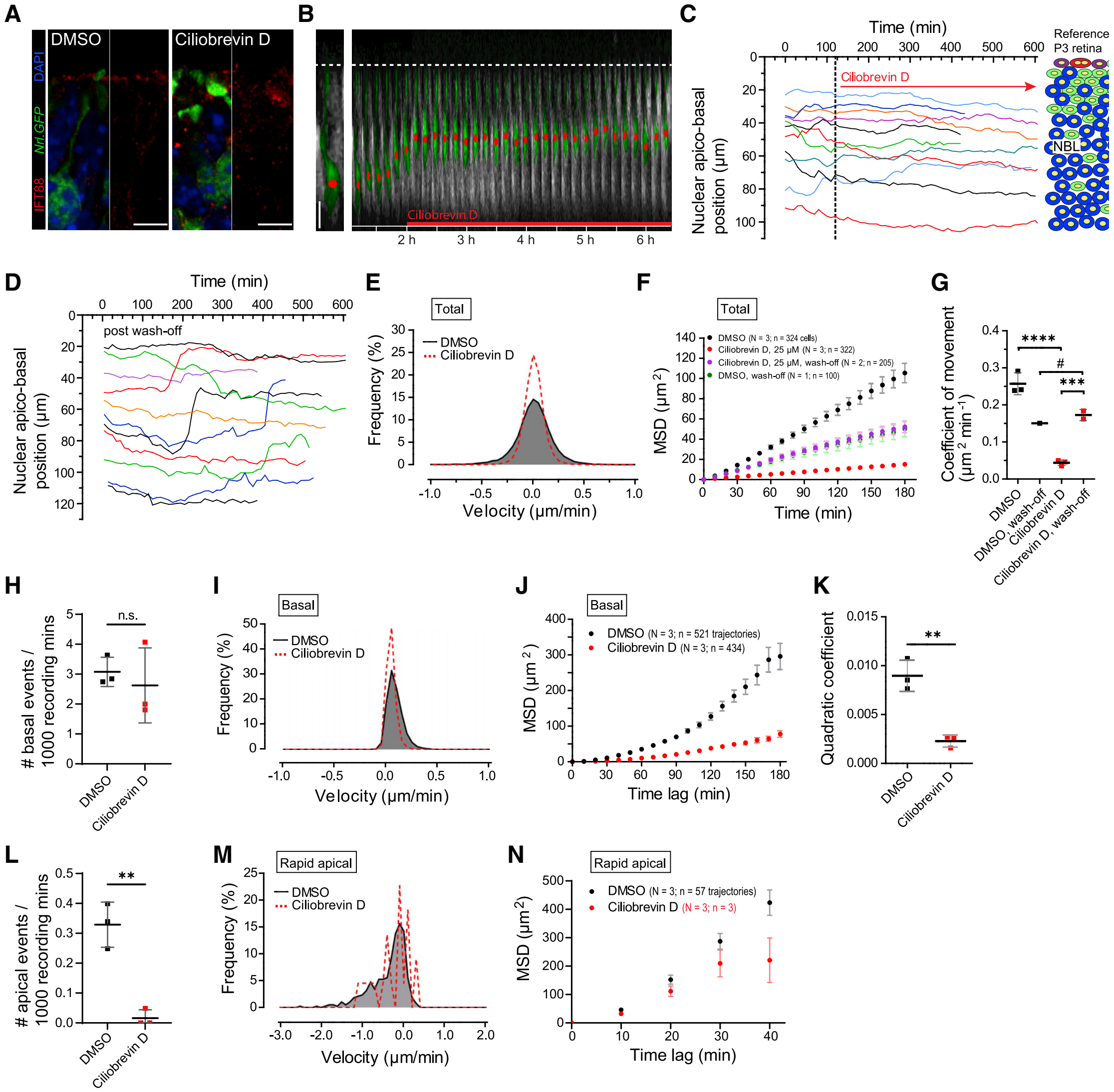

Figure 4. Dynein 1 mediates rapid apical translocation of photoreceptor nuclei

(A) P3 *Nrl.GFP*$^{+/+}$ retina (green), counterstained with DAPI (blue) following 10-h exposure to 25 µM ciliobrevin D resulting in targeting defects of the ciliary transport protein IFT88 (red).

(B) Time-lapse recording of a segmented rod (green) nucleus (red dot) migrating apico-basally in P3 *Nrl.GFP*$^{+/+}$ retina (grayscale) exposed to ciliobrevin D, added after a control period of 120 min.

(C) Representative, overlaid apico-basal rod nuclear trajectories at P3 in presence of ciliobrevin D after 120 mins.

(D) Representative, overlaid apico-basal trajectories at P3 following 30-min ciliobrevin D treatment and subsequent wash out (4 × 30 min).

(E–G) Effect of ciliobrevin D versus DMSO on velocity distribution (E), MSD profiles (F), and coefficients of movement (G) for total rod nuclear movements, including following respective washouts. Data points represent experimental repeats. Unpaired t test.

(H–K) Effect of ciliobrevin D versus DMSO on frequency of basal events (H), velocity distribution (I), MSD profiles (J), and MSD-profile-derived quadratic coefficients (K) for basal movements.

(L–N) Effect of ciliobrevin D versus DMSO on frequency of apical events (L), velocity distribution (M), and MSD profiles (N). N.B. (N) reflect values from only n = 3 recorded rapid apical movements. Scale bars, 5 µm. Unpaired t tests. *$p < 0.05$; **$p < 0.01$; ***$p < 0.001$, ****$p < 0.0001$; $^{\#}$, no statistical test performed due to insufficient data points. Data show mean ± SEM (F, J, and N). See also Figures S3B and S3D–S3F.

Dynein 1 loss of function results in displaced PRs and disrupted ONL stratification

The existence of energy-costly active nuclear translocation within PRs is striking. A failure to translocate apically might be expected to have significant consequences for ONL lamination. To assess whether impairment of rapid apical nuclear translocation affects ONL stratification, we perturbed dynein 1 function specifically in rod PRs by using conditional RNAi and examined the effects at short and long time intervals.

First, we electroporated a floxed short hairpin RNA (shRNA) construct (Ventura et al., 2004) against *Dync1h1*, which encodes an essential dynein 1 subunit, into P1 *Nrl.Cre*$^{+/-}$ mice (Brightman

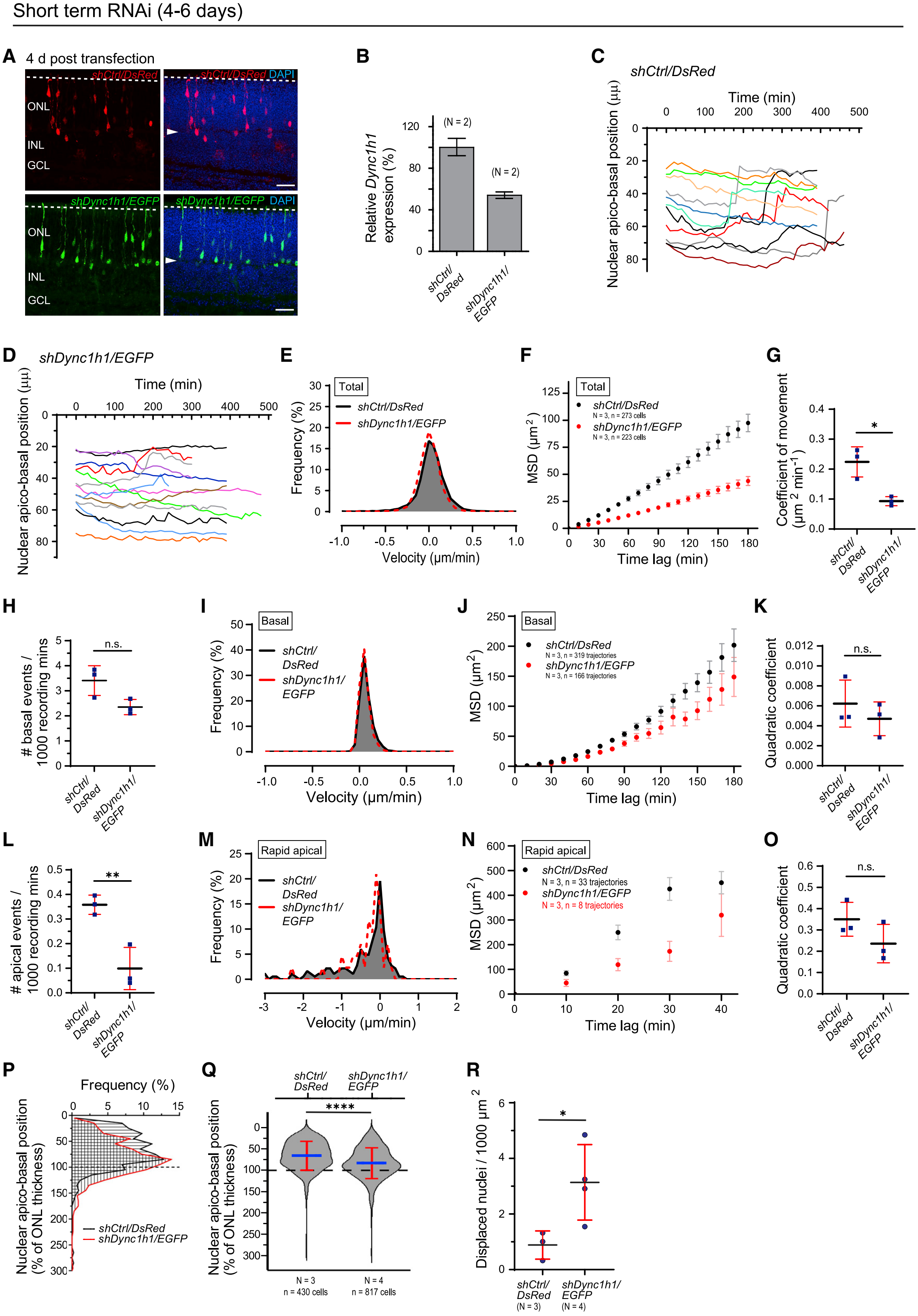

(legend on next page)

et al., 2016). The target sequence for *shDync1h1* was obtained from Tsai et al. (2007) (Figure S5A), and Cre expression is restricted to rod PRs in the *Nrl.Cre*$^{+/-}$ mouse (Figure S5C). Thus, Cre-mediated recombination yields expression of *shDync1h1*, alongside a fluorescent reporter, only in transfected rods. To assess the role of dynein 1 in individual rods, we aimed for relatively sparse transfection (representative transfection levels shown in Figure 5A).

Following electroporation, rod nuclear apico-basal position was assessed by confocal microscopy of fixed tissue (Figures 5A, 5P–5R, S5D, and S5E) and real-time live imaging (Figures 5C–5O) after 4–6 DIV. Real-time imaging showed a reduction in the overall MSD profile of *shDync1h1/EGFP*- versus *shCtrl/DsRed*-expressing cells (*shCtrl/DsRed*: N = 3 retinae, n = 273 cells; *shDync1h1/EGFP*: N = 3, n = 223; Figure 5F), manifesting as a significant reduction in the coefficient of movement, from 0.224 ± 0.050 $\mu m^2\ min^{-1}$ to 0.093 ± 0.015 $\mu m^2\ min^{-1}$ (unpaired t test, $p = 0.013$; Figure 5G). The instantaneous velocity profile remained largely unchanged (Figure 5E). Basally directed nuclear movements were reduced compared to those of the control, but the effect was not significant (Figures 5H–5K and S3H; Table S1; no. of basally directed events/1,000 recording mins: 3.4 ± 0.6 [*shCtrl/DsRed*] versus 2.4 ± 0.3 [*shDync1h1/EGFP*], unpaired t test, $p = 0.052$; quadratic coefficient: 0.006 ± 0.002 [*shCtrl/DsRed*], 0.005 ± 0.002 [*shDync1h1/EGFP*], unpaired t test, $p = 0.052$). However, *shDync1h1/EGFP* expression significantly reduced the number of rapid apically directed nuclear migration events, from 0.4 ± 0.0 to 0.1 ± 0.1 events/1,000 recording mins (unpaired t test, $p = 0.009$; Figure 5L; Table S1). Kinetic data on instantaneous velocity profile, MSD analysis, and quadratic coefficient are shown for the sake of completeness but are of limited power due to the low remaining event count (Figures 5M–5O).

Consistent with these data, histological analysis showed that nuclei of shCtrl/DsRed^{+ve} rods were distributed throughout the radial extent of the ONL, occasionally extending into the OPL and INL (N = 3, n = 430 cells) (Figures 5A, 5P, and S5D), as also seen in *Nrl.GFP* mice and *Nrl.Cre*$^{+/-}$ *x Ai9* mice of the same or similar age (Figures S5C and S5F). In marked contrast, many nuclei of shDync1h1/EGFP^{+ve} rods were significantly shifted basally and even ectopically displaced into the OPL and INL (N = 4, n = 817 cells) (Figures 5A, 5P, and S5E). *shCtrl/DsRed*- and *shDync1h1/EGFP*-expressing rod nuclei were found at an average apico-basal position corresponding to 66% ± 34% and 83% ± 36% of the developing ONL, respectively (Mann-Whitney test, $p < 0.0001$; Figure 5Q). This finding was accompanied by an increased number of nuclei mis-localized beyond the margins of the nascent ONL, from 0.9 ± 0.5 (equivalent to 14% ± 4% of transfected cells) to 3.1 ± 1.4 nuclei (32% ± 14% of transfected cells) per 1,000 μm^2, as viewed from the apico-basal retinal axis (unpaired t test, $p = 0.044$; Figure 5R).

We next sought to investigate the long-term consequences of dynein 1 perturbation in a large population of rod PRs. AAV2/8 *shDync1h1/EGFP* or AAV2/8 *shCtrl/DsRed* was injected sub-retinally into the eyes of P1 *Nrl.Cre*$^{+/-}$ mice (see Figure 6A for representative transduction levels). To confirm knockdown of *Dync1h1*, we performed qRT-PCR of fluorescence-activated cell sorting (FACS)-sorted DsRed^{+ve} or EGFP^{+ve} PRs from AAV2/8 *shCtrl/DsRed* and AAV2/8 *shDync1h1/EGFP* treated retinae, respectively, at 10 days post-transduction. *Dync1h1* RNA levels were reduced by 46% in cells transduced with AAV2/8 *shDync1h1/EGFP* versus AAV2/8 *shCtrl/DsRed* (Figure 5B; N > 6 pooled retinae), which is very similar to that achieved by Tsai and colleagues using the same RNAi target sequence (Tsai et al., 2007).

Histological analysis at 3-weeks post-viral administration revealed that the nuclei of rods transduced with AAV2/8 *shCtrl/DsRed* were exclusively found within the ONL and were evenly distributed within its depth (N = 3 experimental repeats, 6 eyes per condition in total; Figure 6A, top). Rod BCs, whose nuclei usually locate to the INL, form synaptic connections with rods in the OPL. Accordingly, we detected only minimal fluorescence signal overlap between shCtrl/DsRed^{+ve} rod PRs and rod BCs (PKCα^{+ve}), and this corresponded to their synapses (Figures 6B and 6C, top). In contrast, a significant proportion of nuclei of rods transduced with AAV2/8 *shDync1h1/EGFP* was ectopically located (“basally displaced”) outside the ONL and within the OPL, frequently invading domains usually occupied by rod BC dendrites and nuclei (Figure 6A, bottom). This invasion resulted in increased fluorescence signal overlap between these cell populations (Figures 6B and 6C, bottom).

In areas of high AAV2/8 *shDync1h1/EGFP* viral transduction (see representative images in Figure 6A), the number of basally displaced rod PR nuclei increased from 0.1 ± 0.2 to 6.9 ± 2.5

Figure 5. Short-term dynein 1 loss of function in rods results in impaired rapid apical nuclear translocation and basal displacement

(A) Apico-basal positions of transfected rods expressing *shCtrl/DsRed* (red) or *shDync1h1/EGFP* (green) in *Nrl.Cre*$^{+/-}$ retina following electroporation at P1 and 4 DIV. Arrowhead indicates OPL. See also Figures S5D and S5E.

(B) qRT-PCR analysis of *Dync1h1* expression in rods at 10-days post-*in vivo* administration of AAV2/8 *shDync1h1/EGFP* or AAV2/8 *shCtrl/DsRed* in P0–P1.5 *Nrl.Cre*$^{+/-}$ mice.

(C and D) Representative apico-basal nuclear trajectories of shCtrl/DsRed^{+ve} (C) and shDync1h1/EGFP^{+ve} (D) rods from time-lapse live-imaging experiments. Retinae were electroporated at P1 and cultured 6 DIV.

(E–K) Instantaneous velocity distribution (E), MSD profiles (F), and coefficients of movement (G) of total rod nuclear movements in shDync1h1/EGFP^{+ve} versus shCtrl/DsRed^{+ve} cells. Normalized event count (H), velocity distribution (I), MSD profiles (J), and MSD-profile-derived quadratic coefficients (K) of basally directed rod nuclear movements. See also Figure S3H.

(L–P) Normalized event count (L), velocity distribution (M), MSD profiles (N), and MSD-profile-derived quadratic coefficients (O) of rapid apically directed rod nuclear movements. N.B. (N) and (O) reflect values from n = 8 recorded rapid apical movements. See also Figure S3H. (P) Normalized apico-basal nuclear distribution relative to ONL thickness of shCtrl/DsRed^{+ve} (black) or shDync1h1/EGFP^{+ve} (red) rod cells following electroporation of P1 *Nrl.Cre*$^{+/-}$ retinae and culturing for 4 DIV (fixed tissue).

(Q and R) Apico-basal nuclear positions relative to the thickness of the ONL of rod cells expressing *shCtrl/DsRed* (Q) or *shDync1h1/EGFP* (R). Number of displaced rod PR nuclei per 1,000 μm^2 of retina. Scale bar, 25 μm (A). Mann-Whitney test, unpaired t test; *$p < 0.05$, **$p < 0.01$, ***$p < 0.001$, ****$p < 0.0001$.

CellPress
OPEN ACCESS

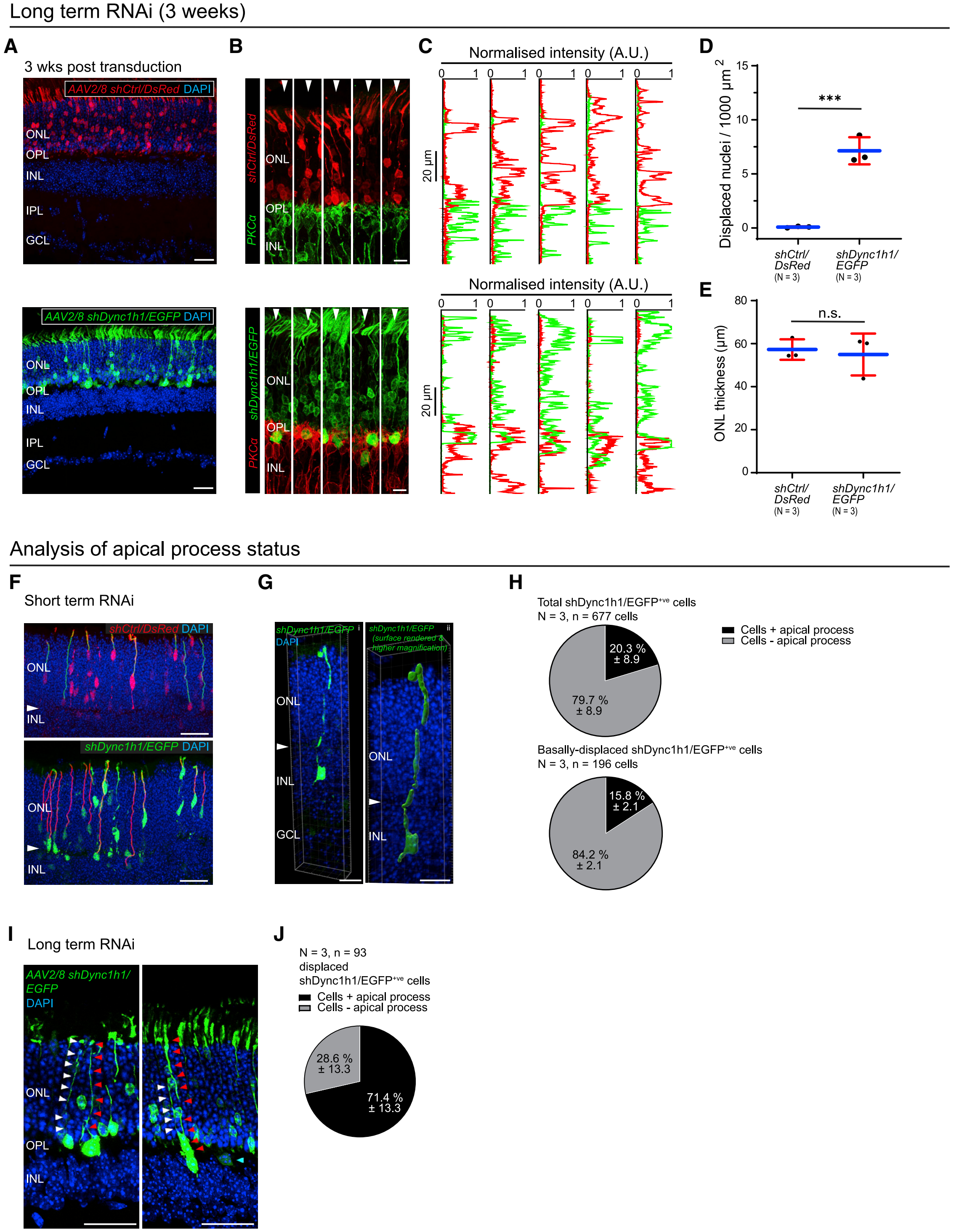

Figure 6. Long-term dynein 1 loss of function in rods results in ectopically located photoreceptors and impaired retinal lamination

(A) Virally transduced (AAV2/8) rods in 3-week-old *Nrl.Cre*$^{+/-}$ retina expressing *shCtrl/DsRed* (top panel, red) or *shDync1h1/EGFP* (bottom panel, green). Virus administered at P1.

(B) PKCα (green/red) in 3-week-old retina with virally transduced rods expressing *shCtrl/DsRed* (red) or *shDync1h1/EGFP* (green).

(C) Representative vertical intensity line profile (position indicated by white arrowheads in B).

(D) Number of displaced rod PR nuclei per 1,000 μm^2 of retina.

(E) ONL thickness.

(legend continued on next page)

nuclei per mm^2 of retina (as viewed from the apico-basal retinal axis; Mann-Whitney test, $p = 0.0006$; Figure 6D). No significant reductions in ONL thickness were seen over the time frame examined (*shCtrl/DsRed*: 57.3 ± 4.8 μm versus *shDync1h1/EGFP*: 55.0 ± 9.7 μm; unpaired t test, $p = 0.730$), indicating that displacement does not bring about widespread cell death (Figure 6E).

We sought to formally exclude the possibility that failure to reposition within the nascent ONL may result from a loss of apical attachment and delamination rather than nuclear motility defects. Differences in the levels of GFP and dsRed mean that often fluorescence signal is detectable only in the soma; whether this reflects a lack of an apical process or sub-threshold fluorescence levels was therefore not distinguishable (Figures 5A, S5D, and S5E). However, 3D reconstruction and apical process tracing of individual shCtrl/DsRed^{+ve} and shDync1h1/EGFP^{+ve} cells at 4 DIV post-electroporation at P1 showed that at least a proportion of cells exhibited apical processes, regardless of displacement status (Figures 6F and 6G). Looking only at those shDync1h1/EGFP^{+ve} cells that were basally displaced beyond the ONL, 16% ± 2% presented a clearly visible apical process (Figure 6H, bottom panel). This is similar to the proportion of total apical process bearing shDync1h1/EGFP^{+ve} cells (20% ± 9%; Figure 6H, top panel; unpaired t test, n.s.). Hence, the presence/absence of a detectable apical process did not correlate with basal displacement status, making it unlikely that apical detachment is required for basal displacement. Similarly, in 3-week-old mice receiving AAV2/8 *shDync1h1/EGFP* at P1, most (71% ± 13%) analyzed basally displaced shDync1h1/EGFP^{+ve} cells retained an apical process (Figures 6I and 6J; N = 3 experimental repeats, n = 93 cells). Thus, although we cannot completely rule out the possibility that some of either *shDync1h1/EGFP*$^{+ve}$ or *shCtrl/DsRed*$^{+ve}$ cells lose their apical process, this is not a prerequisite for basal displacement.

Dynein 1 loss of function in rod PRs impairs correct synapse formation

We next considered whether rods whose nuclei were basally displaced into and beyond the OPL following *Dync1h1* knockdown also exhibited synaptic abnormalities. First, we stained AAV2/8 shRNA-treated retinae for the pre-synaptic ribbon synapse marker ribeye and the rod BC marker PKCα. As expected, in AAV2/8 *shCtrl*-treated retinae, ribeye expression is confined to a band at the level of the OPL (Figure 7A), presenting as a single horseshoe-shaped ribeye structure per reporter-labeled pre-synaptic bouton (N = 3 retinae, n = 93 cells; Figures 7B and 7E–7H; Video S10). In contrast, in retinae treated with AAV2/8 *shDync1h/EGFP*, ribeye staining was frequently displaced into the INL (Figure 7C). Of note, the number of ribeye structures per labeled cell significantly increased, from 1.0 ± 0.1 in shCtrl/DsRed^{+ve} controls to 3.1 ± 2.2 in basally displaced shDync1h/EGFP^{+ve} cells (N = 3 retinae, n = 40 cells; Mann-Whitney test, $p < 0.0001$; Figures 7D–7F; Video S11), although some displaced cells lacked ribeye structures altogether (Figure 7N; Video S12). In shCtrl/DsRed^{+ve} rods, 100% ± 0.0% ribeye staining presented as the classic "horseshoe" shape (Schmitz et al., 1996) versus 31.3% ± 29.5% in shDync1h1/EGFP^{+ve} cells, with the remainder being punctate in appearance (Mann-Whitney test, $p < 0.0001$; Figure 7G).

Rods displaced into the OPL/INL showed heterogeneity with respect to their basal process; many lacked any discernible basal process (Figure 7D), whereas others extended processes laterally or even basally, into the INL (Figures 7K, 7L, and S5G). In keeping with these observations, the cellular location of ribeye was shifted from inside a clearly defined pre-synaptic bouton in shCtrl/DsRed^{+ve} cells (bouton/process: 96.8% ± 17.8%; soma: 3.2% ± 17.8%) to a predominantly somatic location in shDync1h1/EGFP^{+ve} cells (bouton/process: 15.6% ± 27.8%, soma: 84.4% ± 27.8%; Mann-Whitney test, $p < 0.0001$; Figure 7H).

We next investigated whether these ectopic rod pre-synaptic structures retained the ability to associate with post-synaptic BC dendrites by staining retinae for the post-synaptic marker mGluR6, as well as ribeye. This process should typically result in a 1:1 apposition between horseshoe-shaped ribeye staining and punctate mGluR6 staining with a mean distance of 0.51 μm between these markers (Akiba et al., 2019). This finding was indeed the case for shCtrl/DsRed^{+ve} rods (Figures 7I and 7O). However, the probability of ribeye/mGluR6 apposition significantly decreased, from 1.0 ± 0 in shCtrl/DsRed^{+ve} rods to 0.5 ± 0.4 in basally displaced shDync1h1/EGFP^{+ve} cells (one-way ANOVA, $p < 0.001$; Figures 7J–7O). Furthermore, within shDync1h1/EGFP^{+ve} cells, punctate-shaped ribeye structures had a significantly lower mGluR6 apposition probability (0.4 ± 0.5) than those of horseshoe-shaped ribeye (0.7 ± 0.4) (one-way ANOVA, $p < 0.01$; Figure 7O).

Given dynein's role in numerous trafficking events, it is possible that the loss of ribeye localization relates to some other dynein-dependent process, rather than nuclear basal displacement per se. We thus also examined ribeye/mGluR6 distribution in those shDync1h1/EGFP^{+ve} cells that remained within the ONL. Indeed, 96% ± 0% of shDync1h1/EGFP^{+ve} rods retained within the ONL and extending a visible basal protrusion also exhibited normal synapses in terms of ribeye foci location, shape, and number, as well as mGluR6 opposition (N = 3 retinae, n = 137 cells; Figures 7P and 7Q); this value is virtually identical to that of *shCtrl/DsRed*-expressing rod cells, which exhibited 100% ± 0% correct synapses (N = 3 retinae, n = 93 cells) (Figures 7F–7I and 7O). These data suggest that nuclear displacement beyond the ONL due to *Dync1h1* knockdown is likely to lead to significant perturbations in synaptic organization.

(F) Apical process tracking of shCtrl/DsRed^{+ve} (red) or shDync1h1/EGFP^{+ve} (green) rods in *Nrl.Cre*$^{+/-}$ retina following electroporation at P1 and culturing for 4 DIV; arrowheads indicate OPL. Only processes that extended from the soma to the apical limit of the ONL were tracked.

(G) Basally displaced shDync1h1/EGFP^{+ve} rod PR. Native fluorescence signal and 3D surface rendering are shown. Arrowheads indicate OPL.

(H) Apical process status in total (top) and displaced shDync1h1/EGFP^{+ve} rod population (bottom).

(I) Apical processes of selected, displaced shDync1h1/EGFP^{+ve} rod cells (arrowheads) at 3 weeks post-transduction at P1. For some rods, apical processes were not reliably detectable (cyan arrowhead, right panel).

(J) Apical process status among displaced, shDync1h1/EGFP^{+ve} rods. Scale bars, 25 μm (A, F, and I), 10 μm (G), 5 μm (B). Unpaired t test; ***$p < 0.001$.

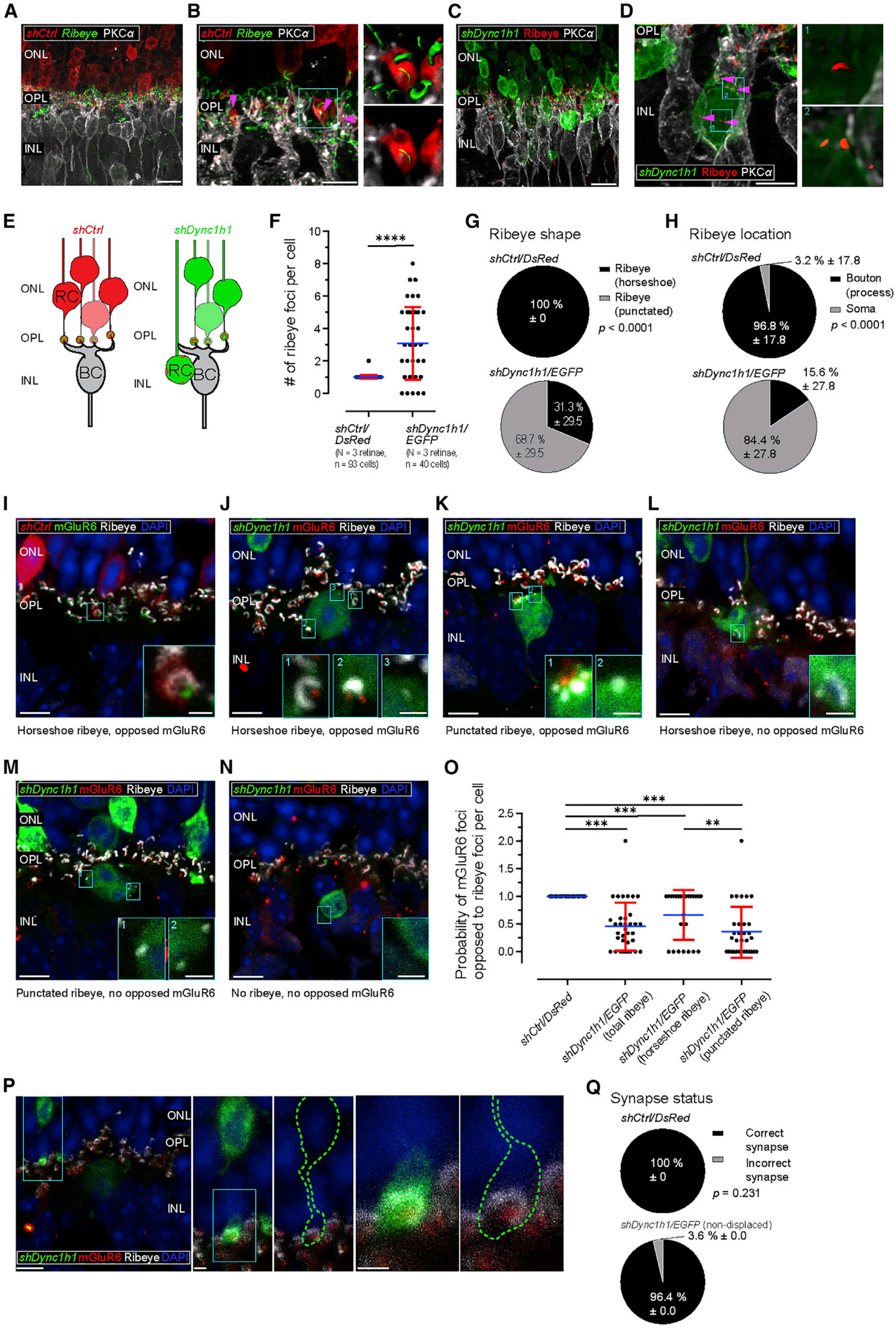

(legend on next page)

DISCUSSION

The PR layer of the mouse retina is many nuclei deep, and its correct development facilitates the formation of correctly located synaptic contacts with the inner retina, which in turn ensures proper retinal function (Dick et al., 2003; Haeseleer et al., 2004; Maddox et al., 2015; Mansergh et al., 2005). Here, we show that ONL lamination requires PR motility featuring repeated, apically directed movements to retain PRs within the ONL. Rapid apical nuclear translocation is an active process, driven by the MT-associated dynein 1 motor protein. Dynein 1 disruption in rod PRs impaired apical translocation and displaced many nuclei beyond the normal limits of the ONL, into the OPL and INL. For those basally displaced cells, this was also associated with impaired synapse formation. This study thus identifies repeated rapid apical nuclear translocation as a mechanism important for correct stratification of post-mitotic PR neurons within the ONL of the mammalian retina.

There are notable similarities in the nuclear kinetic movement profiles of post-mitotic PRs and neuroepithelial progenitor cells undergoing interkinetic nuclear migration (INM), for which nuclei translocate within the elongated progenitor cell in sync with the cell cycle (Kosodo et al., 2011; Leung et al., 2011; Norden et al., 2009; Strzyz et al., 2015; Tsai et al., 2010). Both undergo repeated cycles of rapid, apical translocations and slower, basally directed movements, with the obvious distinction that PR nuclear movement is not linked to the cell cycle. Whether repeated rapid apical nuclear translocation, with or without cell division, is common to other apically located cell populations in stratified epithelia is yet to be explored. Nevertheless, our study identifies repeated apically directed translocations as a novel pattern of somal translocation in post-mitotic neurons (for comprehensive reviews of neuronal migration see Cooper, 2013; Marín et al., 2010).

Apically directed nuclear translocations have been ascribed to different molecular mechanisms, depending on the species studied (reviewed in Kosodo, 2012). In the relatively short (apico-basal length) neuroepithelia of the zebrafish, actomyosin contractile mechanisms drive apical translocation of RPCs (Norden et al., 2009; Strzyz et al., 2016)· However, in the thicker neuroepithelium of the developing mammalian cortex, MTs and associated protein motors (dynein 1 and kinesins) are more important (Baffet et al., 2015; Hu et al., 2013; Kosodo et al., 2011; Tsai et al., 2005, 2010). Here, we find that post-mitotic PRs use MT-associated dynein 1 to power rapid apical nuclear translocation. This result is in keeping with findings from genetic studies in zebrafish and *Drosophila* in which the nuclei of PRs defective in *dctn1* (a dynein 1 co-factor) (Tsujikawa et al., 2007; Whited et al., 2004) or *dync1h1* (Insinna et al., 2010) were also displaced toward the basal edge of the ONL. Similarly, mammalian cone nuclei failed to migrate apically in mice that overexpressed *KASH*, which disrupts LINC complexes and may uncouple dynein from the nuclear envelope (Razafsky et al., 2012).

We hypothesize that PR nuclear oscillations are a consequence, at least partially, of proximal RPC INM motions. The purpose behind the apical translocation of G2-phase progenitor cell nuclei has been a matter of considerable debate but appears to be connected to correct lamination and epithelial integrity (Spear and Erickson, 2012; Strzyz et al., 2015). However, in a spatially constrained sheet with an apical limit, it also basally displaces the nuclei of neighboring cells due to steric crowding at the apical margin. *In silico* simulations of progenitor cell INM to demonstrate that all basal displacements can be exclusively caused by apically directed nuclear translocations support this notion (Kosodo et al., 2011). Razafsky and colleagues speculated that the basal displacement of cones observed following disruption of LINC complexes resulted either from an atypical kinesin-based, LINC-independent mechanism or was the result of passive basal

Figure 7. Displaced rod photoreceptors form atypical synaptic contacts

(A) Low magnification of AAV2/8 virally transduced rods in 3-week-old *Nrl.Cre*$^{+/-}$ retina expressing *shCtrl/DsRed* (red) with pre-synaptic marker (ribeye, green) and PKCα (BCs; grayscale) immunolabeling.
(B) High magnification of *shCtrl/DsRed*$^{+ve}$ rods (red) and ribeye (green) relative to PKCα-labeled BCs (grayscale). Typical rod synaptic bouton shown in magnified panels (ribeye was 3D surface rendered and segmented in bottom panel).
(C) Low magnification of virally transduced shDync1h1/EGFP^{+ve} rods (green) with Ribeye (red) and PKCα (grayscale) immunolabeling.
(D) High magnification of rod soma (green) displaced in INL exhibiting atypical perinuclear ribeye (red; magenta arrowheads). Rod BCs were stained for PKCα (grayscale). ROIs show horseshoe-shaped (1) and punctated ribeye (2) with 3D surface rendering (red).
(E) Schematic representation of cellular and synaptic organization between shCtrl/DsRed^{+ve} and shDync1h1/EGFP^{+ve} rods and rod BCs including ribeye (green).
(F) Number of ribeye foci per cell. Data points represent individual cells.
(G) Ribeye shape (horseshoe versus punctate) frequency.
(H) Ribeye location (bouton/process versus soma) frequency.
(I) AAV2/8 *shCtrl/DsRed*-treated retina (red) stained for ribeye (grayscale) and mGluR6 (green) showing correct synaptic labeling (inserted panel shows high magnification of ROI).
(J–N) *shDync1h1/EGFP*-treated retinae (green) stained for ribeye (grayscale) and mGluR6 (red) exhibiting different categories of pre/post-synaptic marker labeling, as follows: horseshoe-shaped ribeye/mGluR6 opposition (J), punctate ribeye/mGluR6 apposition (K), unapposed horseshoe-shaped ribeye (L), unapposed punctate ribeye (M), and completely absent ribeye (N) (inserts show high magnification of ROIs; note that only the first panels show representative examples of the intended categories).
(O) Probability of ribeye/mGluR6 apposition in shCtrl/DsRed^{+ve} versus shDync1h1/EGFP^{+ve} cells. For shDync1h1/EGFP^{+ve} cells, a further distinction was made between total, horseshoe-shaped, and punctated ribeye foci.
(P) shDync1h1/EGFP^{+ve} cells remaining within the ONL exhibit normal synaptic structures (correct number, location, and shape of ribeye foci, as well as mGluR6 opposition). Boxed regions of interest were magnified to highlight representative cell (green outline).
(Q) Synapse status (correct number, location, and shape of ribeye foci, as well as mGluR6 opposition) in shCtrl/DsRed^{+ve} and non-displaced shDync1h1/EGFP^{+ve} rod cells. Scale bars, 10 μm (A and C), 5 μm (B, D, I–N, and P), and 1 μm (I–N and P magnified panels). Mann-Whitney test (F–H), one-way ANOVA (O). $^{**}p < 0.01$, $^{***}p < 0.001$, $^{****}p < 0.0001$.

displacement by neighboring cells (Razafsky et al., 2012). Our data are consistent with the latter. With respect to frequency, it is reasonable to posit that rod oscillations are time locked with RPC cell cycle length, which lengthens during development (30 h at P1, ~40 h by P5) (Young, 1985). The declining mitotic rates with progressing retinogenesis may reduce the requirement for rod apical translocations until both ultimately come to a halt around P10. In keeping with these time frames, we determined that rod PR nuclei undergo a rapid apical translocation event at least once every 50 h at P3–P4. Indirect support for this model comes from our data showing that blocking actomyosin constrictions with blebbistatin, an established inhibitor of mitotic cytokinesis (Straight et al., 2003), attenuated the average travel distance of basally directed rod nuclear translocation events. However, further studies are required to fully define the extent to which dividing RPCs exert basally displacing forces on PRs.

Conversely, we identified dynein 1 to be a key driver of PR nuclear apical translocation. Short-term *Dync1h1* knockdown led to impaired rapid apical translocation events and a shift of affected rod nuclei to more basal locations. Long-term *Dync1h1* knockdown resulted in significant basal displacement of rod PRs, of which many accumulated basal to the ONL, leading to disrupted stratification. Importantly, basally displaced cells typically retain their apical attachment, supporting the hypothesis that displacement arises from a failure to migrate apically rather than from apical detachment. We can only speculate why displaced PRs were not displaced even further into the retina but suggest that it may be due to the network of horizontal cell neurites that already exists in the presumptive OPL (Huckfeldt et al., 2009).

Basal displacement was accompanied by impaired synapse formation, as seen by mis-localization of pre-synaptic ribeye and a reduction of correctly apposed post-synaptic mGluR6. Conversely, those shDync1h1-EGFP^{+ve} cells remaining in the ONL exhibited normal synaptic labeling. This finding suggests that rod PR synaptic architecture is affected not because of dynein disruption per se but because of nuclear displacement beyond the ONL. We cannot completely exclude the possibility that these synaptic abnormalities arise from other dynein-dependent processes and/or differences in the extent of dynein knockdown in individual cells, and this will be an important area of future study. It will also be important to ascertain the effect of these atypical synaptic contacts on visual function.

Another key area for future study will be to determine whether and how cell extrinsic cues from neighboring cells or the extracellular matrix interact with the intrinsic mechanisms of nuclear movement. Our data show that the onset of apical movement is not predetermined to occur at a certain depth within the NBL. Interactions between PRs and their local surroundings may provide a combination of cues that are integrated into the decision to initiate nuclear translocation, as suggested in other systems (Elias et al., 2007; Famulski et al., 2010; Marín et al., 2010). Rod BCs could provide such instructive signals for rod PRs, at least at later stages of development. Sarin and colleagues showed that the OPL manifests within the extent of the nascent ONL in the developing mouse retina, initially resulting in a number of displaced rod PRs (Sarin et al., 2018). They proposed that BC-derived Wnt5a/5b correctly guides rod PR nuclei and axons to the correct location. This idea would be consistent with our findings and those reported in other systems (Witze et al., 2008). Dopaminergic signaling represents another instructive signal. In response to light, intrinsically photosensitive RGCs stimulate dopaminergic amacrine cells to engage in dopaminergic signaling with cone PRs, promoting correct cone nuclear enrichment at the apical limit of the ONL (Tufford et al., 2018) presumably, based on our findings, in a dynein-1-dependent manner. These and other, currently undescribed, signaling mechanisms may jointly provide the cues necessary for PR lamination.

In conclusion, we report a previously undescribed pattern of movement for post-mitotic neurons in stratified epithelia, namely, repeated, rapid apically directed nuclear translocation. We propose that this movement serves to enrich and retain neurons within a given layer and, in the case of the mammalian retina, ensure correct stratification of the ONL.

STAR★METHODS

Detailed methods are provided in the online version of this paper and include the following:

- KEY RESOURCES TABLE
- RESOURCE AVAILABILITY
  - Lead contact
  - Materials availability
  - Data and code availability
- EXPERIMENTAL MODEL AND SUBJECT DETAILS
  - Mice
  - Cell lines
- METHOD DETAILS
  - Molecular reagents and plasmid design
  - Both constructs were cloned into a pD10 plasmid backbone
  - Recombinant adeno-associated virus (AAV) production
  - AAV sub-retinal injection
  - *Ex vivo* retinal electroporation
  - *Ex vivo* retinal explant culture
  - Retinal dissociation and FACS
  - qRT-PCR
  - Immunohistochemistry
  - Microscopy
  - Analysis of displaced nuclei
  - Analysis of synapses
  - Cell tracking methodology
  - Analyses of nuclear motion
- QUANTIFICATION AND STATISTICAL ANALYSIS

SUPPLEMENTAL INFORMATION

Supplemental information can be found online at https://doi.org/10.1016/j.celrep.2021.109461.

ACKNOWLEDGMENTS

We thank Prof. M. Carandini for assistance with data analysis, O. Semenyuk at the IoO vector production facility for technical support, L. Abelleira-Hervas and

J. Hoke for animal husbandry, and the UCL Cell and Developmental Biology Imaging Unit for imaging assistance. We thank A. Kalargyrou for help with maintaining explants and Nrl.Cre$^{+/-}$ lines and M. Rizzi for constructive criticism of earlier drafts of the manuscript. The graphical abstract was created using BioRender.

This work was supported by the Royal Society (UF120046 and RG080398), Moorfields Eye Charity (ST1209J, E170004A, R150032A, and R180005A), Fight for Sight (1448/1449), RP Fighting Blindness, Medical Research Council UK (mr/j004553/1), and Wellcome Trust (082217). N.D.A. was a UCL Grand Challenge PhD student and Fight for Sight-funded post-doctoral research associate. K.M.W.-C., P.V.W., and M.R.R. were Wellcome Trust/MRC PhD students.

AUTHOR CONTRIBUTIONS

Conceptualization, N.D.A., K.M.W.-C., and R.A.P.; methodology, N.D.A., K.M.W.-C., and R.A.P.; software, N.D.A. and M.R.R.; validation, N.D.A. and K.M.W.-C.; formal analysis, N.D.A., M.R.R., and P.V.W.; investigation, N.D.A., K.M.W.-C., and P.V.W.; resources, R.R.A. and R.A.P.; writing – original draft, N.D.A. and R.A.P.; writing – review & editing, N.D.A., A.J.S., R.R.A., and R.A.P.; funding acquisition, R.R.A. and R.A.P.; supervision, R.A.P.

DECLARATIONS OF INTERESTS

The authors declare no competing interests

Received: March 18, 2019
Revised: November 19, 2019
Accepted: July 9, 2021
Published: August 3, 2021

REFERENCES

Akiba, R., Matsuyama, T., Tu, H.-Y., Hashiguchi, T., Sho, J., Yamamoto, S., Takahashi, M., and Mandai, M. (2019). Quantitative and Qualitative Evaluation of Photoreceptor Synapses in Developing, Degenerating and Regenerating Retinas. Front. Cell. Neurosci. *13*, 16.

Akimoto, M., Cheng, H., Zhu, D., Brzezinski, J.A., Khanna, R., Filippova, E., Oh, E.C.T., Jing, Y., Linares, J.-L., Brooks, M., et al. (2006). Targeting of GFP to newborn rods by Nrl promoter and temporal expression profiling of flow-sorted photoreceptors. Proc. Natl. Acad. Sci. USA *103*, 3890–3895.

Anderson, M.J., and Ter Braak, C.J.F. (2003). Permutation tests for multi-factorial analysis of variance. J. Stat. Comput. Simul. *73*, 85–113.

Baffet, A.D., Hu, D.J., and Vallee, R.B. (2015). Cdk1 Activates Pre-mitotic Nuclear Envelope Dynein Recruitment and Apical Nuclear Migration in Neural Stem Cells. Dev. Cell *33*, 703–716.

Berg, H.C. (1993). Random Walks in Biology. (Princeton University Press).

Brightman, D.S., Razafsky, D., Potter, C., Hodzic, D., and Chen, S. (2016). Nrl-Cre transgenic mouse mediates loxP recombination in developing rod photoreceptors. Genesis *54*, 129–135.

Chow, R.W.-Y., Almeida, A.D., Randlett, O., Norden, C., and Harris, W.A. (2015). Inhibitory neuron migration and IPL formation in the developing zebrafish retina. Development *142*, 2665–2677.

Cooper, J.A. (2013). Cell biology in neuroscience: mechanisms of cell migration in the nervous system. J. Cell Biol. *202*, 725–734.

Dick, O., tom Dieck, S., Altrock, W.D., Ammermüller, J., Weiler, R., Garner, C.C., Gundelfinger, E.D., and Brandstätter, J.H. (2003). The presynaptic active zone protein bassoon is essential for photoreceptor ribbon synapse formation in the retina. Neuron *37*, 775–786.

Donovan, S.L., and Dyer, M.A. (2006). Preparation and square wave electroporation of retinal explant cultures. Nat. Protoc. *1*, 2710–2718.

Edqvist, P.-H.D., and Hallböök, F. (2004). Newborn horizontal cells migrate bi-directionally across the neuroepithelium during retinal development. Development *131*, 1343–1351.

Elias, L.A.B., Wang, D.D., and Kriegstein, A.R. (2007). Gap junction adhesion is necessary for radial migration in the neocortex. Nature *448*, 901–907.

Famulski, J.K., Trivedi, N., Howell, D., Yang, Y., Tong, Y., Gilbertson, R., and Solecki, D.J. (2010). Siah regulation of Pard3A controls neuronal cell adhesion during germinal zone exit. Science *330*, 1834–1838.

Ferguson, L.R., Dominguez, J.M., II, Balaiya, S., Grover, S., and Chalam, K.V. (2013). Retinal Thickness Normative Data in Wild-Type Mice Using Customized Miniature SD-OCT. PLoS One *8*, e67265.

Firestone, A.J., Weinger, J.S., Maldonado, M., Barlan, K., Langston, L.D., O'Donnell, M., Gelfand, V.I., Kapoor, T.M., and Chen, J.K. (2012). Small-molecule inhibitors of the AAA+ ATPase motor cytoplasmic dynein. Nature *484*, 125–129.

Fridolfsson, H.N., and Starr, D.A. (2010). Kinesin-1 and dynein at the nuclear envelope mediate the bidirectional migrations of nuclei. J. Cell Biol. *191*, 115–128.

Fridolfsson, H.N., Ly, N., Meyerzon, M., and Starr, D.A. (2010). UNC-83 coordinates kinesin-1 and dynein activities at the nuclear envelope during nuclear migration. Dev. Biol. *338*, 237–250.

Gao, G.-P., Alvira, M.R., Wang, L., Calcedo, R., Johnston, J., and Wilson, J.M. (2002). Novel adeno-associated viruses from rhesus monkeys as vectors for human gene therapy. Proc. Natl. Acad. Sci. USA *99*, 11854–11859.

Gong, S., Zheng, C., Doughty, M.L., Losos, K., Didkovsky, N., Schambra, U.B., Nowak, N.J., Joyner, A., Leblanc, G., Hatten, M.E., and Heintz, N. (2003). A gene expression atlas of the central nervous system based on bacterial artificial chromosomes. Nature *425*, 917–925.

Haeseleer, F., Imanishi, Y., Maeda, T., Possin, D.E., Maeda, A., Lee, A., Rieke, F., and Palczewski, K. (2004). Essential role of Ca2+-binding protein 4, a Cav1.4 channel regulator, in photoreceptor synaptic function. Nat. Neurosci. *7*, 1079–1087.

Herbert, A.L., Fu, M.M., Drerup, C.M., Gray, R.S., Harty, B.L., Ackerman, S.D., O'Reilly-Pol, T., Johnson, S.L., Nechiporuk, A.V., Barres, B.A., and Monk, K.R. (2017). Dynein/dynactin is necessary for anterograde transport of *Mbp* mRNA in oligodendrocytes and for myelination in vivo. Proc. Natl. Acad. Sci. USA *114*, E9153–E9162.

Hoyer-Fender, S. (2010). Centriole maturation and transformation to basal body. Semin. Cell Dev. Biol. *21*, 142–147.

Hsiau, T.H.-C., Diaconu, C., Myers, C.A., Lee, J., Cepko, C.L., and Corbo, J.C. (2007). The cis-regulatory logic of the mammalian photoreceptor transcriptional network. PLoS One *2*, e643.

Hu, D.J., Baffet, A.D., Nayak, T., Akhmanova, A., Doye, V., and Vallee, R.B. (2013). Dynein recruitment to nuclear pores activates apical nuclear migration and mitotic entry in brain progenitor cells. Cell *154*, 1300–1313.

Huckfeldt, R.M., Schubert, T., Morgan, J.L., Godinho, L., Di Cristo, G., Huang, Z.J., and Wong, R.O.L. (2009). Transient neurites of retinal horizontal cells exhibit columnar tiling via homotypic interactions. Nat. Neurosci. *12*, 35–43.

Icha, J., Kunath, C., Rocha-Martins, M., and Norden, C. (2016). Independent modes of ganglion cell translocation ensure correct lamination of the zebrafish retina. J. Cell Biol. *215*, 259–275.

Inoué, S., and Salmon, E.D. (1995). Force generation by microtubule assembly/disassembly in mitosis and related movements. Mol. Biol. Cell *6*, 1619–1640.

Insinna, C., Baye, L.M., Amsterdam, A., Besharse, J.C., and Link, B.A. (2010). Analysis of a zebrafish dync1h1 mutant reveals multiple functions for cytoplasmic dynein 1 during retinal photoreceptor development. Neural Dev. *5*, 12.

Jordan, M.A., and Wilson, L. (2004). Microtubules as a target for anticancer drugs. Nat. Rev. Cancer *4*, 253–265.

Kaewkhaw, R., Kaya, K.D., Brooks, M., Homma, K., Zou, J., Chaitankar, V., Rao, M., and Swaroop, A. (2015). Transcriptome Dynamics of Developing Photoreceptors in Three-Dimensional Retina Cultures Recapitulates Temporal Sequence of Human Cone and Rod Differentiation Revealing Cell Surface Markers and Gene Networks. Stem Cells *33*, 3504–3518.

Kim, J.-W., Yang, H.-J., Brooks, M.J., Zelinger, L., Karakülah, G., Gotoh, N., Boleda, A., Gieser, L., Giuste, F., Whitaker, D.T., et al. (2016). NRL-Regulated

Transcriptome Dynamics of Developing Rod Photoreceptors. Cell Rep. *17*, 2460–2473.

Kosodo, Y. (2012). Interkinetic nuclear migration: beyond a hallmark of neurogenesis. Cell. Mol. Life Sci. *69*, 2727–2738.

Kosodo, Y., Suetsugu, T., Suda, M., Mimori-Kiyosue, Y., Toida, K., Baba, S.A., Kimura, A., and Matsuzaki, F. (2011). Regulation of interkinetic nuclear migration by cell cycle-coupled active and passive mechanisms in the developing brain. EMBO J. *30*, 1690–1704.

Kovács, M., Tóth, J., Hetényi, C., Málnási-Csizmadia, A., and Sellers, J.R. (2004). Mechanism of blebbistatin inhibition of myosin II. J. Biol. Chem. *279*, 35557–35563.

Kracklauer, M.P., Banks, S.M.L., Xie, X., Wu, Y., and Fischer, J.A. (2007). Drosophila klaroid encodes a SUN domain protein required for Klarsicht localization to the nuclear envelope and nuclear migration in the eye. Fly (Austin) *1*, 75–85.

Kruczek, K., Gonzalez-Cordero, A., Goh, D., Naeem, A., Jonikas, M., Blackford, S.J.I., Kloc, M., Duran, Y., Georgiadis, A., Sampson, R.D., et al. (2017). Differentiation and transplantation of embryonic stem cell-derived cone photoreceptors into a mouse model of end-stage retinal degeneration. Stem Cell Reports *8*, 1659–1674.

Leung, L., Klopper, A.V., Grill, S.W., Harris, W.A., and Norden, C. (2011). Apical migration of nuclei during G2 is a prerequisite for all nuclear motion in zebrafish neuroepithelia. Development *138*, 5003–5013.

Maddox, D.M., Collin, G.B., Ikeda, A., Pratt, C.H., Ikeda, S., Johnson, B.A., Hurd, R.E., Shopland, L.S., Naggert, J.K., Chang, B., et al. (2015). A mutation in Syne2 causes early retinal defects in photoreceptors, secondary neurons, and Müller Glia. Invest. Ophthalmol. Vis. Sci. *56*, 3776–3787.

Madisen, L., Zwingman, T.A., Sunkin, S.M., Oh, S.W., Zariwala, H.A., Gu, H., Ng, L.L., Palmiter, R.D., Hawrylycz, M.J., Jones, A.R., et al. (2010). A robust and high-throughput Cre reporting and characterization system for the whole mouse brain. Nat. Neurosci. *13*, 133–140.

Mansergh, F., Orton, N.C., Vessey, J.P., Lalonde, M.R., Stell, W.K., Tremblay, F., Barnes, S., Rancourt, D.E., and Bech-Hansen, N.T. (2005). Mutation of the calcium channel gene Cacna1f disrupts calcium signaling, synaptic transmission and cellular organization in mouse retina. Hum. Mol. Genet. *14*, 3035–3046.

Marín, O., Valiente, M., Ge, X., and Tsai, L.-H. (2010). Guiding neuronal cell migrations. Cold Spring Harb. Perspect. Biol. *2*, a001834.

Matsuda, T., and Cepko, C.L. (2004). Electroporation and RNA interference in the rodent retina in vivo and in vitro. Proc. Natl. Acad. Sci. USA *101*, 16–22.

Meijering, E., Jacob, M., Sarria, J.-C.F., Steiner, P., Hirling, H., and Unser, M. (2004). Design and validation of a tool for neurite tracing and analysis in fluorescence microscopy images. Cytometry A *58*, 167–176.

Merriam, E.B., Millette, M., Lumbard, D.C., Saengsawang, W., Fothergill, T., Hu, X., Ferhat, L., and Dent, E.W. (2013). Synaptic regulation of microtubule dynamics in dendritic spines by calcium, F-actin, and drebrin. J. Neurosci. *33*, 16471–16482.

Morgan, J.L., Dhingra, A., Vardi, N., and Wong, R.O.L. (2006). Axons and dendrites originate from neuroepithelial-like processes of retinal bipolar cells. Nat. Neurosci. *9*, 85–92.

Norden, C., Young, S., Link, B.A., and Harris, W.A. (2009). Actomyosin is the main driver of interkinetic nuclear migration in the retina. Cell *138*, 1195–1208.

Panda, D., Daijo, J.E., Jordan, M.A., and Wilson, L. (1995). Kinetic stabilization of microtubule dynamics at steady state in vitro by substoichiometric concentrations of tubulin-colchicine complex. Biochemistry *34*, 9921–9929.

Patterson, K., Molofsky, A.B., Robinson, C., Acosta, S., Cater, C., and Fischer, J.A. (2004). The functions of Klarsicht and nuclear lamin in developmentally regulated nuclear migrations of photoreceptor cells in the Drosophila eye. Mol. Biol. Cell *15*, 600–610.

Picone, R., Ren, X., Ivanovitch, K.D., Clarke, J.D.W., McKendry, R.A., and Baum, B. (2010). A polarised population of dynamic microtubules mediates homeostatic length control in animal cells. PLoS Biol. *8*, e1000542.

Poggi, L., Vitorino, M., Masai, I., and Harris, W.A. (2005). Influences on neural lineage and mode of division in the zebrafish retina in vivo. J. Cell Biol. *171*, 991–999.

Razafsky, D., Blecher, N., Markov, A., Stewart-Hutchinson, P.J., and Hodzic, D. (2012). LINC complexes mediate the positioning of cone photoreceptor nuclei in mouse retina. PLoS One *7*, e47180.

Reidel, B., Goldmann, T., Giessl, A., and Wolfrum, U. (2008). The translocation of signaling molecules in dark adapting mammalian rod photoreceptor cells is dependent on the cytoskeleton. Cell Motil. Cytoskeleton *65*, 785–800.

Rich, K.A., Zhan, Y., and Blanks, J.C. (1997). Migration and synaptogenesis of cone photoreceptors in the developing mouse retina. J. Comp. Neurol. *388*, 47–63.

Ruthardt, N., Lamb, D.C., and Bräuchle, C. (2011). Single-particle tracking as a quantitative microscopy-based approach to unravel cell entry mechanisms of viruses and pharmaceutical nanoparticles. Mol. Ther. *19*, 1199–1211.

Sainath, R., and Gallo, G. (2014). The dynein inhibitor Ciliobrevin D inhibits the bidirectional transport of organelles along sensory axons and impairs NGF-mediated regulation of growth cones and axon branches. Dev. Neurobiol. *75*, 757–777.

Sarin, S., Zuniga-Sanchez, E., Kurmangaliyev, Y.Z., Cousins, H., Patel, M., Hernandez, J., Zhang, K.X., Samuel, M.A., Morey, M., Sanes, J.R., and Zipursky, S.L. (2018). Role for Wnt Signaling in Retinal Neuropil Development: Analysis via RNA-Seq and In Vivo Somatic CRISPR Mutagenesis. Neuron *98*, 109–126.e8.

Schenk, J., Wilsch-Bräuninger, M., Calegari, F., and Huttner, W.B. (2009). Myosin II is required for interkinetic nuclear migration of neural progenitors. Proc. Natl. Acad. Sci. USA *106*, 16487–16492.

Schindelin, J., Arganda-Carreras, I., Frise, E., Kaynig, V., Longair, M., Pietzsch, T., Preibisch, S., Rueden, C., Saalfeld, S., Schmid, B., et al. (2012). Fiji: an open-source platform for biological-image analysis. Nat. Methods *9*, 676–682.

Schmitz, F., Bechmann, M., and Drenckhahn, D. (1996). Purification of synaptic ribbons, structural components of the photoreceptor active zone complex. J. Neurosci. *16*, 7109–7116.

Sedmak, T., and Wolfrum, U. (2011). Intraflagellar transport proteins in ciliogenesis of photoreceptor cells. Biol. Cell *103*, 449–466.

Smiley, S., Nickerson, P.E., Comanita, L., Daftarian, N., El-Sehemy, A., Tsai, E.L., Matan-Lithwick, S., Yan, K., Thurig, S., Touahri, Y., et al. (2016). Establishment of a cone photoreceptor transplantation platform based on a novel cone-GFP reporter mouse line. Sci. Rep. *6*, 22867.

Spear, P.C., and Erickson, C.A. (2012). Interkinetic nuclear migration: a mysterious process in search of a function. Dev. Growth Differ. *54*, 306–316.

Stepanova, T., Slemmer, J., Hoogenraad, C.C., Lansbergen, G., Dortland, B., De Zeeuw, C.I., Grosveld, F., van Cappellen, G., Akhmanova, A., and Galjart, N. (2003). Visualization of microtubule growth in cultured neurons via the use of EB3-GFP (end-binding protein 3-green fluorescent protein). J. Neurosci. *23*, 2655–2664.

Straight, A.F., Cheung, A., Limouze, J., Chen, I., Westwood, N.J., Sellers, J.R., and Mitchison, T.J. (2003). Dissecting temporal and spatial control of cytokinesis with a myosin II Inhibitor. Science *299*, 1743–1747.

Strzyz, P.J., Lee, H.O., Sidhaye, J., Weber, I.P., Leung, L.C., and Norden, C. (2015). Interkinetic nuclear migration is centrosome independent and ensures apical cell division to maintain tissue integrity. Dev. Cell *32*, 203–219.

Strzyz, P.J., Matejcic, M., and Norden, C. (2016). Heterogeneity, Cell Biology and Tissue Mechanics of Pseudostratified Epithelia: Coordination of Cell Divisions and Growth in Tightly Packed Tissues, International Review of Cell and Molecular Biology (Elsevier Inc.).

Suzuki, S.C., Bleckert, A., Williams, P.R., Takechi, M., Kawamura, S., and Wong, R.O.L. (2013). Cone photoreceptor types in zebrafish are generated by symmetric terminal divisions of dedicated precursors. Proc. Natl. Acad. Sci. USA *110*, 15109–15114.

Tanaka, T., Serneo, F.F., Higgins, C., Gambello, M.J., Wynshaw-Boris, A., and Gleeson, J.G. (2004). Lis1 and doublecortin function with dynein to mediate

coupling of the nucleus to the centrosome in neuronal migration. J. Cell Biol. *165*, 709–721.

Tran, P.T., Marsh, L., Doye, V., Inoué, S., and Chang, F. (2001). A mechanism for nuclear positioning in fission yeast based on microtubule pushing. J. Cell Biol. *153*, 397–411.

Troutt, L.L., and Burnside, B. (1988). Microtubule polarity and distribution in teleost photoreceptors. J. Neurosci. *8*, 2371–2380.

Tsai, J.-W., Chen, Y., Kriegstein, A.R., and Vallee, R.B. (2005). LIS1 RNA interference blocks neural stem cell division, morphogenesis, and motility at multiple stages. J. Cell Biol. *170*, 935–945.

Tsai, J.-W., Bremner, K.H., and Vallee, R.B. (2007). Dual subcellular roles for LIS1 and dynein in radial neuronal migration in live brain tissue. Nat. Neurosci. *10*, 970–979.

Tsai, J.-W., Lian, W.-N., Kemal, S., Kriegstein, A.R., and Vallee, R.B. (2010). Kinesin 3 and cytoplasmic dynein mediate interkinetic nuclear migration in neural stem cells. Nat. Neurosci. *13*, 1463–1471.

Tsujikawa, M., Omori, Y., Biyanwila, J., and Malicki, J. (2007). Mechanism of positioning the cell nucleus in vertebrate photoreceptors. Proc. Natl. Acad. Sci. USA *104*, 14819–14824.

Tufford, A.R., Onyak, J.R., Sondereker, K.B., Lucas, J.A., Earley, A.M., Mattar, P., Hattar, S., Schmidt, T.M., Renna, J.M., and Cayouette, M. (2018). Melanopsin Retinal Ganglion Cells Regulate Cone Photoreceptor Lamination in the Mouse Retina. Cell Rep. *23*, 2416–2428.

Ventura, A., Meissner, A., Dillon, C.P., McManus, M., Sharp, P.A., Van Parijs, L., Jaenisch, R., and Jacks, T. (2004). Cre-lox-regulated conditional RNA interference from transgenes. Proc. Natl. Acad. Sci. USA *101*, 10380–10385.

Waldron, P.V., Di Marco, F., Kruczek, K., Ribeiro, J., Graca, A.B., Hippert, C., Aghaizu, N.D., Kalargyrou, A.A., Barber, A.C., Grimaldi, G., et al. (2018). Transplanted Donor- or Stem Cell-Derived Cone Photoreceptors Can Both Integrate and Undergo Material Transfer in an Environment-Dependent Manner. Stem Cell Reports *10*, 406–421.

West, E.L., Gonzalez-Cordero, A., Hippert, C., Osakada, F., Martinez-Barbera, J.P., Pearson, R.A., Sowden, J.C., Takahashi, M., and Ali, R.R. (2012). Defining the integration capacity of embryonic stem cell-derived photoreceptor precursors. Stem Cells *30*, 1424–1435.

Whited, J.L., Cassell, A., Brouillette, M., and Garrity, P.A. (2004). Dynactin is required to maintain nuclear position within postmitotic Drosophila photoreceptor neurons. Development *131*, 4677–4686.

Witze, E.S., Litman, E.S., Argast, G.M., Moon, R.T., and Ahn, N.G. (2008). Wnt5a control of cell polarity and directional movement by polarized redistribution of adhesion receptors. Science *320*, 365–369.

Young, R.W. (1984). Cell death during differentiation of the retina in the mouse. J. Comp. Neurol. *229*, 362–373.

Young, R.W. (1985). Cell proliferation during postnatal development of the retina in the mouse. Brain Res. *353*, 229–239.

Yu, J., Lei, K., Zhou, M., Craft, C.M., Xu, G., Xu, T., Zhuang, Y., Xu, R., and Han, M. (2011). KASH protein Syne-2/Nesprin-2 and SUN proteins SUN1/2 mediate nuclear migration during mammalian retinal development. Hum. Mol. Genet. *20*, 1061–1073.

Zacharias, D.A., Violin, J.D., Newton, A.C., and Tsien, R.Y. (2002). Partitioning of lipid-modified monomeric GFPs into membrane microdomains of live cells. Science *296*, 913–916.

Zhang, X., Lei, K., Yuan, X., Wu, X., Zhuang, Y., Xu, T., Xu, R., and Han, M. (2009). SUN1/2 and Syne/Nesprin-1/2 complexes connect centrosome to the nucleus during neurogenesis and neuronal migration in mice. Neuron *64*, 173–187.

Zolessi, F.R., Poggi, L., Wilkinson, C.J., Chien, C.-B., and Harris, W.A. (2006). Polarization and orientation of retinal ganglion cells in vivo. Neural Dev. *1*, 2.

CellPress
OPEN ACCESS

STAR★METHODS

KEY RESOURCES TABLE

REAGENT or RESOURCE	SOURCE	IDENTIFIER
Antibodies		
Rabbit polyclonal α-tubulin	Abcam	Cat#ab24246, RRID:AB_447954
Goat polyclonal IFT88	Abcam	Cat#ab42497, RRID:AB_778681
Sheep mGluR6	Kind gift from K. Martemyanov	N/A
Rabbit polyclonal PH3	Millipore	Cat#06-570, RRID:AB_310177
Rabbit polyclonal PKCα	Sigmaaldrich	Cat#P4334, RRID:AB_477345
Mouse monoclonal ribeye	BD Biosciences	Cat#612044, RRID:AB_399431
Mouse monoclonal γ-tubulin	Abcam	Cat#ab11316, RRID:AB_297920
Alexa Fluor® 488 goat anti-rabbit	ThermoFisher Scientific	Cat#A32731, RRID:AB_2633280
Alexa Fluor® 546 goat anti-rabbit	ThermoFisher Scientific	Cat#A-11071, RRID:AB_2534115
Alexa Fluor® 633 goat anti-rabbit	ThermoFisher Scientific	Cat#A-21070, RRID:AB_2535731
Alexa Fluor® 488 goat anti-mouse	ThermoFisher Scientific	Cat#A-11001, RRID:AB_2534069
Alexa Fluor® 546 goat anti-mouse	ThermoFisher Scientific	Cat#A-11018, RRID:AB_2534085
Alexa Fluor® 546 donkey anti-goat	ThermoFisher Scientific	Cat#A-11056, RRID:AB_2534103
Alexa Fluor® 488 donkey anti-sheep	ThermoFisher Scientific	Cat#A-11015, RRID: AB_2534082
Alexa Fluor® 546 donkey anti-sheep	ThermoFisher Scientific	Cat#A- 21098, RRID: AB_2535752
Bacterial and virus strains		
α-Select Gold Competent *E. coli*	Bioline	Cat#BIO-85027
AAV2/8 *shDync1h1/EGFP*	This paper	N/A
AAV2/8 *shCtrl/DsRed*	This paper	N/A
Chemicals, peptides, and recombinant proteins		
Blebbistatin	Sigmaaldrich	Cat#B0560
Ciliobrevin D	Millipore	Cat#250401
Demecolcine	Sigmaaldrich	Cat#D7385
DMSO	Sigmaaldrich	Cat#D8418
Taurine	Sigmaaldrich	Cat#T4571
Critical commercial assays		
Papain Dissociation System	Worthington	Cat#LK003153
RNeasy Micro Kit	QIAGEN	Cat#74004
QuantiTect Reverse Transcription Kit	QIAGEN	Cat#205311
Experimental models: Cell lines		
HEK293T cells		RRID:CVCL_0063
Experimental models: Organisms/strains		
Nrl.GFP$^{+/+}$ mice (*B6.Cg-Tg(Nrl-EGFP)1Asw/J*)	kind gift of A. Swaroop	RRID:IMSR_JAX:02 1232
Nrl.Cre$^{+/-}$ mice (*C57BL/6J-Tg(Nrl-cre)1Smgc/J*)	The Jackson Laboratory	RRID:IMSR_JAX:02 8941
Ai9 mice (*B6;129S6-Gt(ROSA)26Sor*$^{tm9(CAG}$*tdTomato)Hze/J*)	The Jackson Laboratory	RRID:IMSR_JAX:007905
Chrnb4.EGFP mice (*Tg(Chrnb4EGFP)CL200Gsat/Mmnc*)	MMRRC	RRID:MMRRC_0002 59-UNC
C57BL/6J mice	Harlan Laboratories	N/A
Oligonucleotides		
Actb F primer (AAGGCCAACCGTGAAAAGAT)	Sigmaaldrich	N/A
Actb R primer (GTGGTACGACCAGAGGCATAC)	Sigmaaldrich	N/A
Dync1h1 F primer (ATGAAGCCCTCCGTCTCTTC)	Sigmaaldrich	N/A
Dync1h1 R primer (GTCAATGTTTTCGTCAGTCCAG)	Sigmaaldrich	N/A

(*Continued on next page*)

Continued		
REAGENT or RESOURCE	SOURCE	IDENTIFIER
Universal probe library probe #56	Roche	Cat# 04688538001
Universal probe library probe #88	Roche	Cat# 04689135001
Recombinant DNA		
pD10 Nrl.EGFP	This paper	N/A
pD10 Nrl.Cent2-DsRed	This paper	Sub-cloned from Addgene plasmid Cat#29523
pD10 Nrl.EB3-tdTomato	This paper	Subcloned from Addgene plasmid Cat#50708
pD10 Nrl.myr/palm-mCherry	This paper	Subcloned from Zacharias et al., 2002
pD10 shDync1h1/EGFP (pD10 mU6.TL-shCtrlTL.shDync1h1 / CMV.FL-pA-FL.EGFP)	This paper	N/A
pD10 shCtrl/DsRed (pD10 mU6.shCtrl / CMV.FL-pAFL.DsRed)	This paper	N/A
AAV8 capsid	Kind gift of A. Nathwani	N/A
pHGTi helper plasmid	Kind gift of A. Nathwani	N/A
Software and algorithms		
GraphPad Prism	GraphPad	https://www.graphpad.com/scientificsoftware/prism/
Fiji/ImageJ	NIH	https://imagej.nih.gov/ij/
IMARIS	Bitplane	http://www.bitplane.com/imaris
MATLAB	Mathworks	https://www.mathworks.com/products/MATLAB.html
Huygens Deconvolution	Scientific Volume Imaging	https://svi.nl/HuygensDeconvolution
Illustrator	Adobe	https://www.adobe.com/Illustrator
Other		
MATLAB code	This Paper	https://github.com/RPearsonLab/Photoreceptor_tracking

RESOURCE AVAILABILITY

Lead contact

Further information and requests for resources and reagents should be directed to and will be fulfilled by the Lead Contact, Rachael Pearson (rachael.pearson @kcl.ac.uk).

Materials availability

All unique/stable reagents generated in this study will be made available on request but may require a payment and/or a completed Materials Transfer Agreement if there is potential for commercial application.

Data and code availability

The imaging data reported in this study cannot be deposited in a public repository because they do not comprise a standardized datatype. Moreover, the authors are undertaking further analysis and any outputs arising from these will be published in due course. To request access, contact the lead author.

All original code has been deposited in the 'Photoreceptor_tracking' Github repository and is publicly available as of the date of publication. URL is listed in the Key resources table.

Any additional information required to re-analyze the data reported in this paper is available from the lead contact upon request.

EXPERIMENTAL MODEL AND SUBJECT DETAILS

Mice

Nrl.GFP$^{+/+}$ mice (2.5kb upstream segment of Nrl gene drives *EGFP* expression; kind gift of A. Swaroop, University of Michigan, USA; bred in-house; RRID:IMSR_JAX:021232) (Akimoto et al., 2006), *Nrl.Cre*$^{+/-}$ mice (1.7kb mouse Nrl promoter drives *Cre* recombinase

expression; kind gift of S. Chen; University of Washington, USA; bred in house as hemizygotes; RRID:IMSR_JAX:028941) (Brightman et al., 2016), Ai9 mice (RRID:IMSR_JAX:007905) (Madisen et al., 2010), *Chrnb4.EGFP* mice (RRID:MMRRC_000259-UNC) (Gong et al., 2003), and wild-type *C57BL/6J* mice (Harlan Laboratories) were used, according to the NC3R ARRIVE guidelines, between embryonic day (E) 16 and P14. Adult mice were 6-8wks of age. Both male and female mice were used in this study without discrimination.

Male and female mice were group housed in the animal facility at University College London on a standard 12-hour light/dark cycle at the same light levels throughout the experimental period. Animals were kept in individually ventilated cages on animal grade wood chip and given access to nesting material and food and water *ad libitum*.

All animal studies were carried out under the Animals (Scientific Procedures) Act 1986 under a project license PPL 70/8120 issued by the UK Government Home Office and conducted in accordance with protocols approved by the Animal Welfare and Ethics Committee of the UCL Institute of Ophthalmology. All animals were killed by cervical dislocation performed by trained personnel (approved under Schedule 1 as a method of humane killing). All efforts were made to minimize the number and suffering of animals used in these experiments.

Cell lines

HEK293T cells (RRID:CVCL_0063; Sex: female) were used for the production of AAV vectors. They were maintained as adherent cell cultures in 15 cm Petri dishes in 20 mL maintenance medium (DMEM (GIBCO, ThermoFisher Scientific) supplemented with 10% fetal bovine serum (GIBCO, ThermoFisher Scientific)) at 37°C and 5% CO_2 in humidified incubators. For passaging, 80% confluent cells were incubated in 0.05% trypsin solution (GIBCO, ThermoFisher Scientific) for 5 min at 37°C and 5% CO_2. Trypsin was subsequently inactivated by the addition of maintenance medium, followed by cell splitting as appropriate.

METHOD DETAILS

Molecular reagents and plasmid design

Nrl promoter-driven expression constructs

EGFP, Cent2-DsRed (Addgene plasmid #29523) (Tanaka et al., 2004), *EB3-tdTomato* (Addgene plasmid # 50708) (Merriam et al., 2013), and *myr/palm-mCherry* (Zacharias et al., 2002) were each subcloned into a pD10 expression and AAV packaging-compatible construct downstream of the *NRL* promoter region. A 2.5 kb segment upstream of the *Nrl* gene was cloned from mouse genome and used as the *NRL* promoter in this study, as per (West et al., 2012)·

shDync1h1/EGFP and shCtrl/DsRed RNAi constructs

To clone the conditional *shDync1h1* RNAi construct pD10 *mU6.TL-shCtrl-TL.shDync1h1 / CMV.FL-pA-FL.EGFP* (abbreviated to *shDync1h1/EGFP)*, a scrambled control short hairpin (target sequence: 5′- GATCGGACACTCCTCATAA-3′) flanked by *TATA-lox* (*TL*) sites designed for conditional shRNA expression from the *mU6* promoter (Ventura et al., 2004) was placed between *mU6* promoter and a short hairpin sequence against *Dync1h1* (target sequence: 5′- AGGCTTTAACCAAGCAGATAA-3′; based on findings by Tsai et al. (2007) who reduced protein levels by 50% in cultured rat neurons) (Figure 6A). This entire conditional RNAi module against *Dync1h1* was synthesized (GeneArt Gene Synthesis, ThermoFisher Scientific). A separate conditional reporter module was cloned into the same plasmid by placing a *poly(A)* sequence flanked by *loxP* sites between *CMV* promoter and *EGFP* open reading frame. Cre enzyme mediates independent recombination events at shRNA and reporter modules. The RNAi control construct pD10 *mU6.shCtrl / CMV.FL-pA-FL.DsRed* (*abbreviated to shCtrl/DsRed*) provides constitutive *shCtrl* expression by placing the scrambled control short hairpin sequence immediately downstream of the *mU6* promoter and conditional reporter expression by placing a *poly(A)* sequence flanked by *loxP* sites between *CMV* promoter and *DsRed* open reading frame.

Both constructs were cloned into a pD10 plasmid backbone

All plasmids were transformed into α-Select Gold Competent *E. coli* (Bioline) and subsequently purified using the QIAGEN plasmid mega kit (QIAGEN).

Recombinant adeno-associated virus (AAV) production

The conditional RNAi (*shDync1h1/EGFP*) and control constructs (*shCtrl/DsRed*), containing AAV-2 inverted terminal repeats (ITRs), were encapsidated into recombinant AAV particles of serotype 8 to produce AAV2/8 *shDync1h1/EGFP* and AAV2/8 *shCtrl/DsRed*. This was achieved using a tri-partite plasmid transfection system on HEK293T cells, as previously described (Gao et al., 2002). Briefly, DNA mix consisting of the three DNA plasmids *shDync1h1/EGFP* or *shCtrl/DsRed,* AAV8 capsid and pHGTi helper plasmid were mixed at a molar ratio of 1:1:3 with PEI transfection reagent (Polysciences; 2.25 μg PEI per 1 μg DNA) in DMEM. The transfection mix was added to 80% confluent HEK293T cells at 50 μg DNA per 15 cm cell culture plate. Three days post transfection, HEK293T cells were harvested in harvesting buffer (140 mM NaCl / 5 mM KCl / 0.7 mM K_2HPO_4 / 3.5 mM $MgCl_2$ / 25 mM Tris base in H_20, pH 7.5). Cells were subsequently lysed by four freeze/thaw/vortex cycles. Viral particles were purified by affinity chromatography on an AVB Sepharose column (GE Healthcare). The eluate was concentrated to a volume of 200 μl using Vivaspin columns (Sartorius AG) to achieve a titer of 5×10^{13} vector genomes (vg)/ml. Viral titers were determined by real-time quantitative PCR using primers specific for the ITRs, as described previously (Kruczek et al., 2017).

AAV sub-retinal injection

For *in vivo* administration, 0.4 μl of viral preparation (2 × 10^{10} vg/eye) were sub-retinally injected into P0-P1.5 *Nrl.Cre*$^{+/-}$ mice anaesthetized on ice prior to injections. Eye lids were surgically opened, pupils were dilated using 1% tropicamide and treated with topical anesthetic (amethocaine). Eyes were protected from dehydration with Viscotears™ (Novartis). Sub-retinal injections were administered under direct visual control through an operating microscope (Zeiss) using a sterile syringe (Hamilton) fitted with a 34 gauge hypodermic bevel-edged needle placed between neural retina and RPE. Mice were subsequently allowed to recover on heat mat before being returned to parent mice.

Ex vivo retinal electroporation

DNA plasmids were transfected by electroporation (Hsiau et al., 2007; Matsuda and Cepko, 2004). For *in vitro* electroporation, retinae from P0 - 2.5 mice were transferred into a 2-mm gap size electroporation cuvette (BTX) containing 1 μg/μl plasmid DNA in PBS. Using a pulse generator (model ECM 830, BTX), the retinae were electroporated with 5 × 30 V square pulses of 50 ms duration and with 950 ms intervals. The retinae were allowed to recover for 5 min each in serum free and subsequently in 5% fetal calf serum (ThermoFisher Scientific) containing media (1:1 DMEM/F-12 with L-glutamine and 15 mM HEPES (ThermoFisher Scientific) supplemented with1 mM Taurine (Sigma)).

Ex vivo retinal explant culture

Following the protocol by Donovan and Dyer (2006) explanted retinae were placed vitread side down on 0.2 μm polycarbonate membranes (Whatman) and cultured at 37°C / 5% CO_2 in a sitting drop of 5% fetal calf serum (ThermoFisher Scientific) containing media (1:1 DMEM/F-12 with L-glutamine and 15 mM HEPES (ThermoFisher Scientific) supplemented with1 mM Taurine (Sigma) for four to ten days. To counteract evaporation, explant culture-containing sitting drops were re-supplied with 50-100 μl fresh media every day.

Retinal dissociation and FACS

Neural retinae were harvested from Nrl.Cre$^{+/-}$ mice 10 days post injection at P0-P1.5 with AAV2/8 *shDync1h1/EGFP* or AAV2/8 *shCtrl/DsRed* by dissection and dissociated using the papain dissociation system (Worthington) according to the manufacturer's instructions. Briefly, retinae were enzymatically dissociated in EBSS / 20 U/ml papain / 1:100 v/v antibiotic/antimycotic at 37°C / 5% CO_2 for 45 mins. The cell suspension was gently triturated with a 200 μL pipette tip, passed through a 70 μm strainer and spun down at 200 g for 5 mins. Cell pellets were resuspended in EBSS / 1 mg/ml ovomucoid protease inhibitor / 100 U/mL DNase I and incubated for 5-10 mins at 37°C / 5% CO_2. The suspension was subsequently layered over an EBSS / 10 mg/ml ovomucoid protease inhibitor solution and centrifuged at 100 g for 5 mins. Finally, cell pellets were resuspended in FACS buffer (EBSS / 1% FCS) prior to sorting on a special order 5-laser BD Influx Cell Sorter (BD Biosciences). FACS sorted GFP$^+$ or DsRed$^+$ cells were collected in EBSS / 50% FCS.

qRT-PCR

RNA was extracted from FACS sorted GFP$^+$ or DsRed$^+$ cells using the RNeasy Micro Kit (QIAGEN) and reverse-transcribed using the QuantiTect Reverse Transcription Kit (QIAGEN). qPCR assays were performed using the 2x FastStart TaqMan® Probe Mastermix (Roche) in conjunction with the Universal ProbeLibrary system technology (Roche). Primers for the target (*Dync1h1*; F: ATGAAGCC CTCCGTCTCTTC, R: GTCAATGTTTTCGTCAGTCCAG) and endogenous reference control markers (*Actb*; F: AAGGCCAACCGT GAAAAGAT, R: GTGGTACGACCAGAGGCATAC) were designed and probes were chosen (probe # 88 and 56 respectively) according to recommendations by the Universal Probe Library Design Center (Roche). Reaction mixes were prepared according to the table below:

Reagent	Stock concentration	Volume (μl)	Final concentration
Forward primer	20 μM	0.2	200 nM
Reverse primer	20 μM	0.2	200 nM
Probe	10 μM	0.2	100 nM
cDNA	1.5 x dilution from reverse transcription reaction	5	variable
PerfeCTa® qPCR FastMix® II, Low ROX™	2 x	10	1 x
ddH_2O	-	4.4	-
Final volume		20 μl	

Reaction mixes were loaded onto MicroAmp® Optical 96-Well Reaction Plates (Applied Biosystems). qRT-PCR was performed on an ABI Prism 7900HT Fast Real-Time PCR Sequence Detection System (Applied Biosystems) set to perform the following program:

PCR step	Temperature (°C)	Duration	# of cycles
Activation of FastStart Taq DNA polymerase	95	10 min	1x
Denaturation	95	15 s	50x
Annealing/Extension	60	1 min	

Immunohistochemistry

For immunohistochemistry, eyes or retinal tissue were fixed in 4% (wt/vol) paraformaldehyde (Sigma) for at least 30 mins prior to cryopreservation in 20% (wt/vol) sucrose (Sigma) overnight (o/n). After embedding in OCT (Pyramid Innovation), tissues were sectioned at 18 μm thickness on a Bright OTF5000 cryostat (Bright Instruments Co Ltd). Tissue sections were washed with PBS (pH 7.4), blocked in PBS supplemented with 5% (vol/vol) goat or donkey serum (Bio-Rad), 1% (wt/vol) BSA (Sigma) and 0.1% (vol/vol) Triton X-100 (Sigma). Primary antibodies were applied to sections over night at 4°C, followed by washes in PBS and subsequent application of secondary antibodies for 2-4 hr at room temperature (goat/donkey Alexa Fluor antibodies with 488, 546 or 633 fluorophores as appropriate (Thermofisher). Nuclei were counterstained with 4′,6-Diamidin-2-phenylindol (DAPI; Sigma; shown in blue in all confocal images) at 1 μg/ml. Primary antibodies used in this study were: α-tubulin (Abcam, ab24246, RRID:AB_447954, 1:500), mGluR6 (kind gift from K. Martemyanov, 1:200) PKCα (Sigma, P4334, RRID:AB_477345, 1:10.000), PH3 (Millipore, 06-570, RRID:AB_310177, 1:250), ribeye (BD Biosciences, 612044, RRID:AB_399431, 1:100) and γ-tubulin (Abcam, ab11316, RRID:AB_297920, 1:100). Negative controls omitted the primary antibody.

Microscopy

Live imaging by time lapse 2-photon microscopy

Live retinae were flattened and whole mounted with the PR side up onto a 0.45 μm MF-Millipore nitrocellulose membrane (Millipore). Placed in DMEM^{gfp}-2 live imaging medium (Evrogen), time-lapse recordings of retinae were performed on a Leica SP8 upright confocal laser scanning microscope (Leica) equipped with 25x or 40x water-immersion objectives (NA = 0.95 and 0.8 respectively) as well as Leica photomultiplier tube/avalanche photo diode hybrid HyD detectors (Leica). The multiphoton laser source (Coherent) was tuned to a wavelength of 900 nm for the excitation of GFP and DsRed. All recordings were made at 37°C / 5% CO_2. For image acquisition, *xyzt* image series were captured at a resolution of 512x512, at a step size of 1 μm and at 15 s (EB3-tdTomato experiments) or 10 min intervals (nuclear motility experiments). For pharmacological investigations, retinae were treated with 25 μM Blebbistatin (Sigma), 25 μM Ciliobrevin D (Millipore) or 45 nM Demecolcine (Sigma) after a 2-hour control period. 0.1% DMSO was used as vehicle control. For the Ciliobrevin D washout experiment, explanted retinae were drug-treated for 30 min at 37°C / 5% CO_2 followed by 4x 30 min washes in imaging medium at 37°C / 5% CO_2 prior to imaging. *xyzt* image series were processed and registered in Fiji/ImageJ (Schindelin et al., 2012).

Confocal microscopy of fixed specimens

Fixed tissues were imaged using a Leica TCS SPE confocal laser scanning microscope fitted with 40x (NA = 1.15) and 63x (NA = 1.3) objectives and photomultiplier tubes to detect fluorescence emission. For image acquisition, *xyz* confocal stacks were captured at a resolution of 1024 × 1024 pixels and at a step size of 0.25 - 1 μm, as appropriate. Selected images were deconvolved using Huygens Deconvolution software (Scientific Volume Imaging). For image presentation in figures, color labels were placed on top of black background surface for increased readability for Figures 6A, 6F, 7 (all image panels), S5F, and S5G.

Analysis of displaced nuclei

For the analysis of rod nuclear displacement on confocal micrographs of fixed retinae in the shRNA expression experiments (*shCtrl/DsRed or shDync1h1/EGFP*), the apico-basal position of the centers of transfected/transduced rod cell nuclei was assessed, using Fiji/ImageJ. To quantify the position of each individual rod soma relative to the ONL, we defined the ONL as the radial width (based on DAPI labeling) from the apical-most to the basal-most DAPI labeling at the exact tangential position of each rod cell within its associated field of view and retina. Apico-basal positions between apical and basal ONL margins were classified as correctly localized, whereas positions more basal than the basal ONL margin were classified as basally-displaced. This methodology was applied to both short term (electroporation into P0 retinal explants and tissue harvest after 4 days of *in vitro* culture) and long term shRNA expression experiments (*in vivo* AAV injection at P0 and tissue harvest at 3 weeks post injection).

Analysis of synapses

For the analysis of synapses between *shCtrl/DsRed* or *shDync1h1/EGFP*-expressing rods and bipolar cells, retinae were immunolabelled for ribeye (pre-synaptic) and mGluR6 (post-synaptic). Image analysis was performed in Fiji/ImageJ. For *shCtrl/DsRed*$^+$ PRs, analysis was restricted to cells with clearly traceable and connected soma, axon, and synaptic bouton, while only those *shDync1h1/EGFP*$^+$ rod cells with a basally displaced nucleus were considered.

The ribeye confocal signal was processed to increase the signal:noise ratio and to clearly segment true pre-synaptic structures (usually horseshoe-shaped), similarly to Akiba et al. (2019)). This was achieved by using the "enhance contrast" function, applying

a bandpass filter to remove small particle noise, followed by the "smooth" function to obtain a more homogeneous ribeye signal within each region and finally by applying a size filter on manually thresholded images within the "analyse particles" function to further remove small particle background noise. Ribeye structures clearly located within *shCtrl/DsRed* or *shDync1h1/EGFP*-expressing rods were counted and classified according to shape (horseshoe versus punctated) and subcellular location (synaptic bouton/process versus soma).

The mGluR6 confocal signal was similarly processed by utilizing the "enhance contrast" function, followed by applying a size filter on manually thresholded images within the "analyse particles" function to remove small particle background noise; finally, the "smooth" function was used to obtain a more homogeneous mGluR6 signal within each region. Opposition between pre- and post-synaptic termini was evident when pre-synaptic ribeye and post-synaptic mGluR6 structures were within a distance of 0.51 μm (Akiba et al., 2019).

Cell tracking methodology

To track the movement of rod cell bodies in time lapse recordings, the spot tracking tool within IMARIS software (Bitplane) was applied to the *xyzt* time-lapse image series and set to identify the following traceable features of immature rod somata: i) GFP^{+ve}, ii) ellipsoid in shape, iii) ellipsoid dimensions of 7.5 μm. Computer-generated positional information over time (*x, y, z,* and *t*) was manually verified.

To track location and dimension parameters of the apical processes of rod PRs in confocal micrographs from fixed retinae the Fiji/ImageJ plug-in NeuronJ was used (Meijering et al., 2004).

Analyses of nuclear motion

Kinetic analyses on nuclear trajectories were performed in Microsoft Excel (Microsoft) and with custom routines in MATLAB (Mathworks). We restricted our analyses to one-dimensional nuclear trajectories along the apico-basal tissue axes (*zt*), since motility along this dimension was predominantly observed compared with two-dimensional lateral motility (*xyt*) (see Figure S1B). The following velocity criteria were used for the different movement types observed in this study: $x \leq -10$ μm in 30 min (rapid apical), $x \geq 15$ μm in ≥ 2hrs (basally-directed). The criterion for rapid apical movement was formed to reflect the rapid apical translocations observed in this study. To distinguish between persistent basally-directed movement and other periods of no net movement (stochastic), we used a threshold of movement greater than 2x apico-basal rod somal lengths, typically at 7.5 μm (15 μm) in a 2 hr period. To compare oscillation, rapid apical, and basal event frequency, event counts were normalized by the cumulative recording minutes (sum of all trajectory durations (mins) within a given retina). This was necessary due to differences in the number and duration of trajectories between retinae. Absolute event counts were normalized as follows: normalized event count = absolute event count / cumulative recording minutes x 1000 recording minutes.

Instantaneous velocity calculations: average velocities were first obtained by determining nuclear positional changes relative to the apical tissue margin in consecutive time frames. These were subsequently transformed into instantaneous velocity measurements in the *i*th frame by dividing by the recording interval between frames (δt = 10 min) according to:

$$v(i\delta t) = \frac{\delta z}{\delta t} = \frac{z((i+1)\delta t) - z(i\delta t)}{\delta t},$$

where $z(i\delta t)$ and $z((i+1)\delta t)$ denote the *z* position of a given nucleus at time frames $i\delta t$ and the consecutive time frame $(i+1)\delta t$.

Mean squared displacement: we used the mean squared displacement (MSD) as a function of elapsed time as a measure of the average distance traveled by PR nuclei (Ruthardt et al., 2011). MSD analysis was analogously used previously when describing nuclear motilities of dividing epithelial progenitor cells undergoing INM (Leung et al., 2011; Norden et al., 2009). MSD values were calculated by taking the average of squared displacements displayed by a nucleus within a given trajectory over successively increasing time-intervals. This was followed by further averaging the trajectories of populations of cells. To this end, the following equation was applied:

$$MSD(\Delta t) = \frac{1}{N-n} \sum_{i=1}^{N-n} [z((i+n)\delta t) - z(i\delta t)]^2$$

where $z(i\delta t)$ and $z((i+n)\delta t)$ are the *z* positions of a given nucleus at time frames $i\delta t$ and $(i+n)\delta t$ respectively, *n* is an integer representing the time interval between those positions and *N* is the total number of time points within the time-lapse recording. To quantify changes in the MSD, the MSD data points were subjected to curve fitting. For particles subject to non-directional motion, the MSD is a linear function of elapsed time Δt,

$$MSD = 2RD\Delta t$$

with a one-dimensional slope of 2*R*D = 2D, where *R* is the dimensionality (in the present study, *R* = 1) and where D is the coefficient of movement. The coefficient *D* was used to quantitively compare MSDs of non-directional and *total* rod nuclear translocations (since *total* rod nuclear translocations were predominantly non-directional, they were also subjected to linear function curve fitting). For rapid apically- and basally-directed movements, the MSD displays a quadratic dependence on elapsed time, which is indicative

CellPress
OPEN ACCESS

of active and/or directed movement (Berg, 1993; Ruthardt et al., 2011). The MSD profiles of rapid apical and basally-directed translocations were curve fitted in GraphPad Prism® software (GraphPad Software Inc., RRID:SCR_002798) with the quadratic function

$$y = ax^2$$

to obtain the quadratic factor *a* as numerical representative of curve steepness, which was used to quantitatively compare MSDs of rapid apical and basally-directed rod nuclear translocations.

QUANTIFICATION AND STATISTICAL ANALYSIS

All means are stated ± standard deviation, unless otherwise specified. N = number of eyes and n = number of cells analyzed. For qualitative and quantitative histological assessments, at least 3 eyes from independent animals were used per group. For time lapse studies, given the required duration of the live imaging experiments, only one retina from a given animal was imaged in any one experimental run and is considered an independent sample. We used GraphPad Prism® software (GraphPad Software Inc.) and custom routines in MATLAB (Mathworks) for statistical analyses. D'Agostino and Pearson test was used to assess the normality of datasets. For statistical tests involving one independent variable to be compared between 2 groups we used the unpaired t test and Mann-Whitney test for normally and non-normally distributed datasets respectively. For the comparison of one independent variable between > 2 groups, we used 1-Way ANOVA with Tukey's multiple comparison test. For statistical tests involving two independent variables we used two-way ANOVA with a post hoc permutation test including Monte Carlo randomization (Anderson and Ter Braak, 2003). Significance was accepted at $p \leq 0.05$.

Cell Reports, Volume *36*

Supplemental information

Repeated nuclear translocations underlie photoreceptor positioning and lamination of the outer nuclear layer in the mammalian retina

Nozie D. Aghaizu, Katherine M. Warre-Cornish, Martha R. Robinson, Paul V. Waldron, Ryea N. Maswood, Alexander J. Smith, Robin R. Ali, and Rachael A. Pearson

	DMSO	Demecolcine	Blebbistatin	Ciliobrevin D	*shCtrl/DsRed*	*shDync1h1/EGFP*
Normalised # basal events	3.1 ± 0.5	3.4 ± 0.6	2.7 ± 0.7	2.6 ± 1.3	3.4 ± 0.6	2.4 ± 0.3
Normalised # apical events	0.3 ± 0.1	0.2 ± 0.1	0.4 ± 0.2	0.0 ± 0.0 **	0.4 ± 0.0	0.1 ± 0.1 **

Table S1. Frequency of rapid apically- and basally-directed events.

Related to Figure 1-5.

Normalised number of observed rapid apically- and basally-directed events per 1000 total recording minutes in DMSO control or drug exposed live P3 retinal explants subjected to time lapse microscopy. Event count was normalised by cumulative recording minutes (see Methods). *shCtrl/DsRed* and *shDync1h1/EGFP* data were obtained from live imaged *Nrl.Cre*$^{+/-}$ retinae that had previously been electroporated at P1 and cultured for 6DIV. **$p < 0.01$; unpaired t test

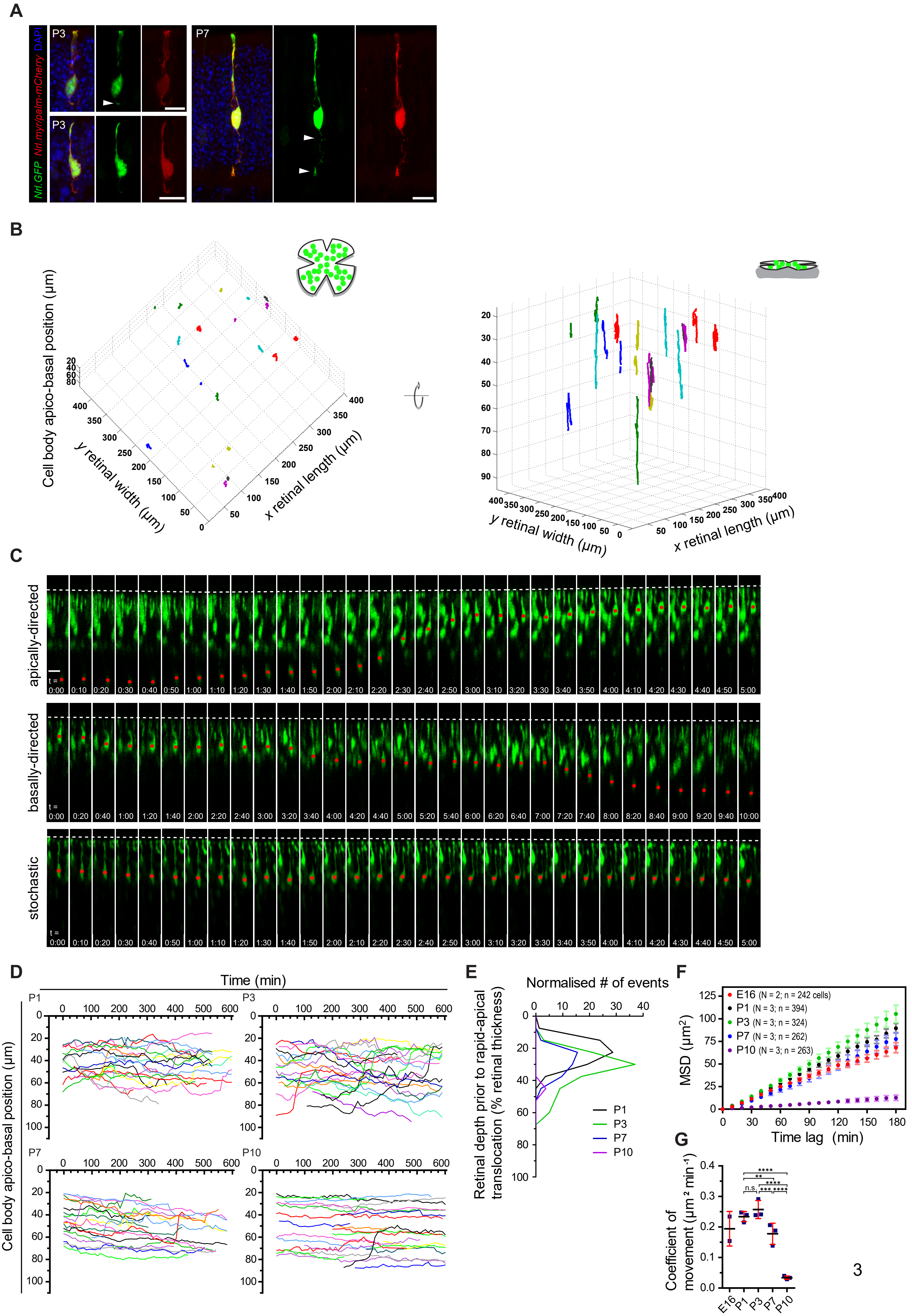
A
Nrl.GFP Nrl.myr/palm-mCherry DAPI
P3
P3
P7
B
Cell body apico-basal position (µm)
y retinal width (µm)
x retinal length (µm)
C
apically-directed
basally-directed
stochastic
t =
D
Time (min)
P1
P3
P7
P10
Cell body apico-basal position (µm)
E
Normalised # of events
Retinal depth prior to rapid-apical translocation (% retinal thickness)
P1
P3
P7
P10
F
E16 (N = 2; n = 242 cells)
P1 (N = 3; n = 394)
P3 (N = 3; n = 324)
P7 (N = 3; n = 262)
P10 (N = 3; n = 263)
MSD (µm²)
Time lag (min)
G
Coefficient of movement (µm² min⁻¹)
n.s.
E16
P1
P3
P7
P10

3

Supplementary Fig. S1. Rod photoreceptor nuclei undergo apico-basal translocations during retinogenesis.

Related to Fig. 1.

(A) Expression of cytoplasmic *Nrl.EGFP* (green) and membrane-targeting *Nrl.myr/palm-mCherry* (red) in wild-type retinae counterstained with DAPI (blue) to visualise the basal process (arrowhead). Electroporation was performed at P1. At P3, the basal process was not always present and could be of variable length. Where a basal process was detected, membrane label (*myr/palm-mCherry*) was frequently evident in absence of cytoplasmic EGFP (compare top vs bottom panels). At P7, both cytoplasmic and membrane label were visible in most cases (right panels). **(B)** Live-imaged field of view (*xyzt*) of a flatmounted P3 *Nrl.GFP*$^{+/+}$ retina showing the same representative nuclear trajectories as viewed from different angles. **(C)** Time lapse recording of a manually segmented rod (green) nucleus (red dot) migrating along the apico-basal cellular axis in a P3 *Nrl.GFP*$^{+/+}$ retina. Apical (top) and basal (middle) movements, as well as non-directional periods with no net positional change (bottom series) are shown. See also Supplementary movies S2-4. Dotted line indicates apical retinal limit. **(D)** Representative rod PR apico-basal nuclear trajectories observed in P1, P3, P7, and P10 retinae superimposed in a single *zt* plot. Each individual trajectory represents an individual nuclear trajectory. **(E)** Distribution of apico-basal starting positions of rapid apical nuclear translocations at P1-P10 expressed as % of retinal thickness. Retinal thickness measurements were obtained from fixed reference retinae at each of the time points. **(F)** Mean squared displacement (MSD) profiles of pooled rod nuclear translocations observed from E16-P10. **(G)** Coefficients of movement (directly proportional to the slopes of the MSD curves; see methods) are shown for each time point from E16-P10. Experimental repeats, each containing a whole set of nuclear trajectories, were collapsed down to individual data points (blue). Time point E16 only consists of 2 experimental repeats and was thus not included in the statistical analysis. Scale bars, 10 µm. 2-way ANOVA with post-hoc permutation test. n.s., not significant; *$p < 0.05$; **$p < 0.01$; ***$p < 0.001$; ****$p < 0.0001$. Data show mean ± SEM (**F**).

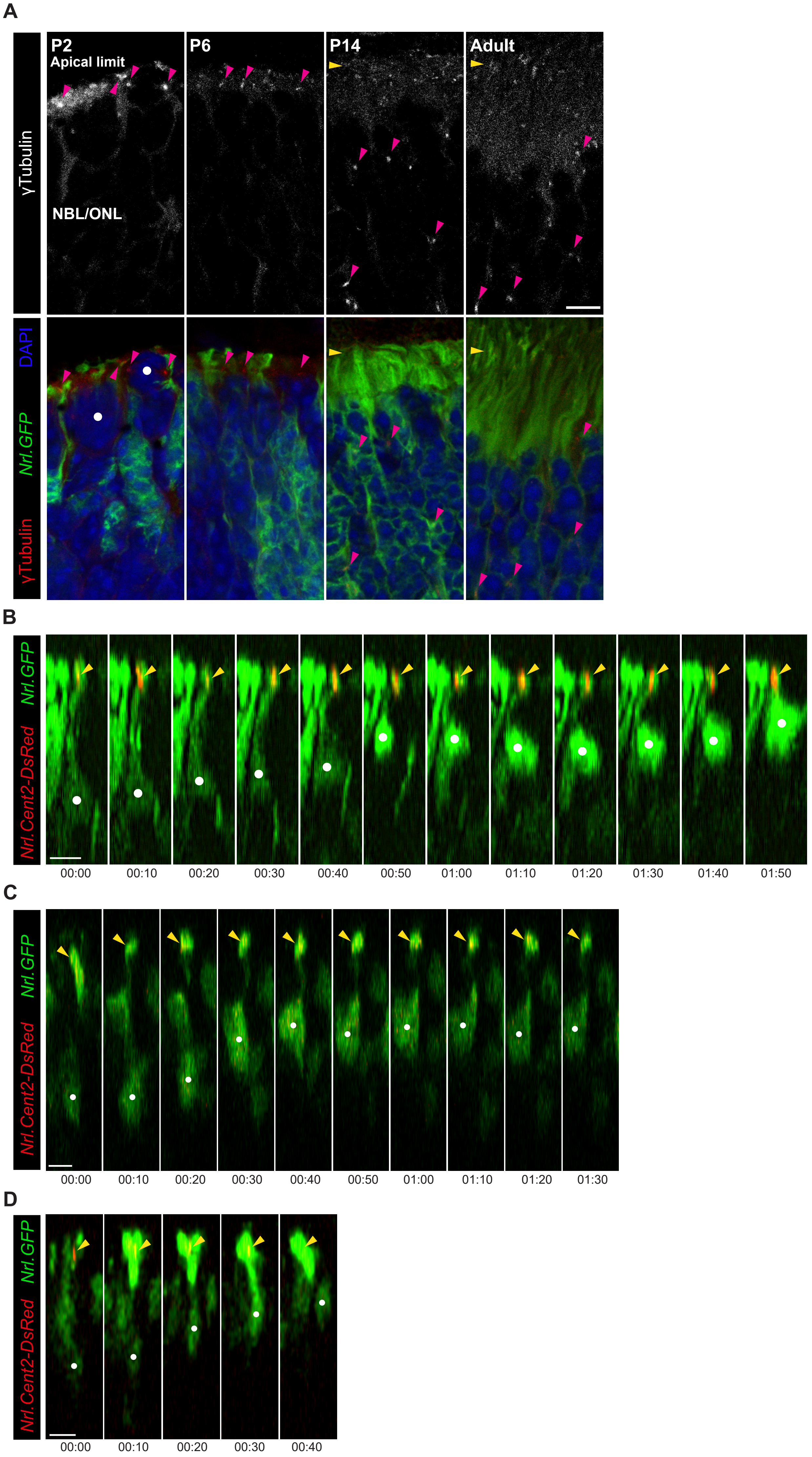

A
P2
Apical limit
NBL/ONL
P6
P14
Adult
γTubulin
DAPI
Nrl.GFP
γTubulin
B
Nrl.GFP
Nrl.Cent2-DsRed
00:00
00:10
00:20
00:30
00:40
00:50
01:00
01:10
01:20
01:30
01:40
01:50
C
Nrl.GFP
Nrl.Cent2-DsRed
00:00
00:10
00:20
00:30
00:40
00:50
01:00
01:10
01:20
01:30
D
Nrl.GFP
Nrl.Cent2-DsRed
00:00
00:10
00:20
00:30
00:40

Supplementary Fig. S2. Rod nuclear motility does not require motile centrosomes.

Related to Fig. 2.

(A) γ-tubulin (grey in top panel, red in lower panel) labels all centrosomes. At P2, labelling is restricted to the apical margin but becomes more dispersed by P8 as post-mitotic neurons migrate away. **(B-D)** Time lapse series of explanted P1 *Nrl.GFP*$^{+/+}$ retinae (green) electroporated with *Nrl.Cent2-DsRed* (red) and cultured *in vitro* for 3 days. The centrosomes remain at the apical tissue limit (yellow arrowhead) during rapid apically-directed nuclear translocation (white dot). Scale bars, 5 µm.

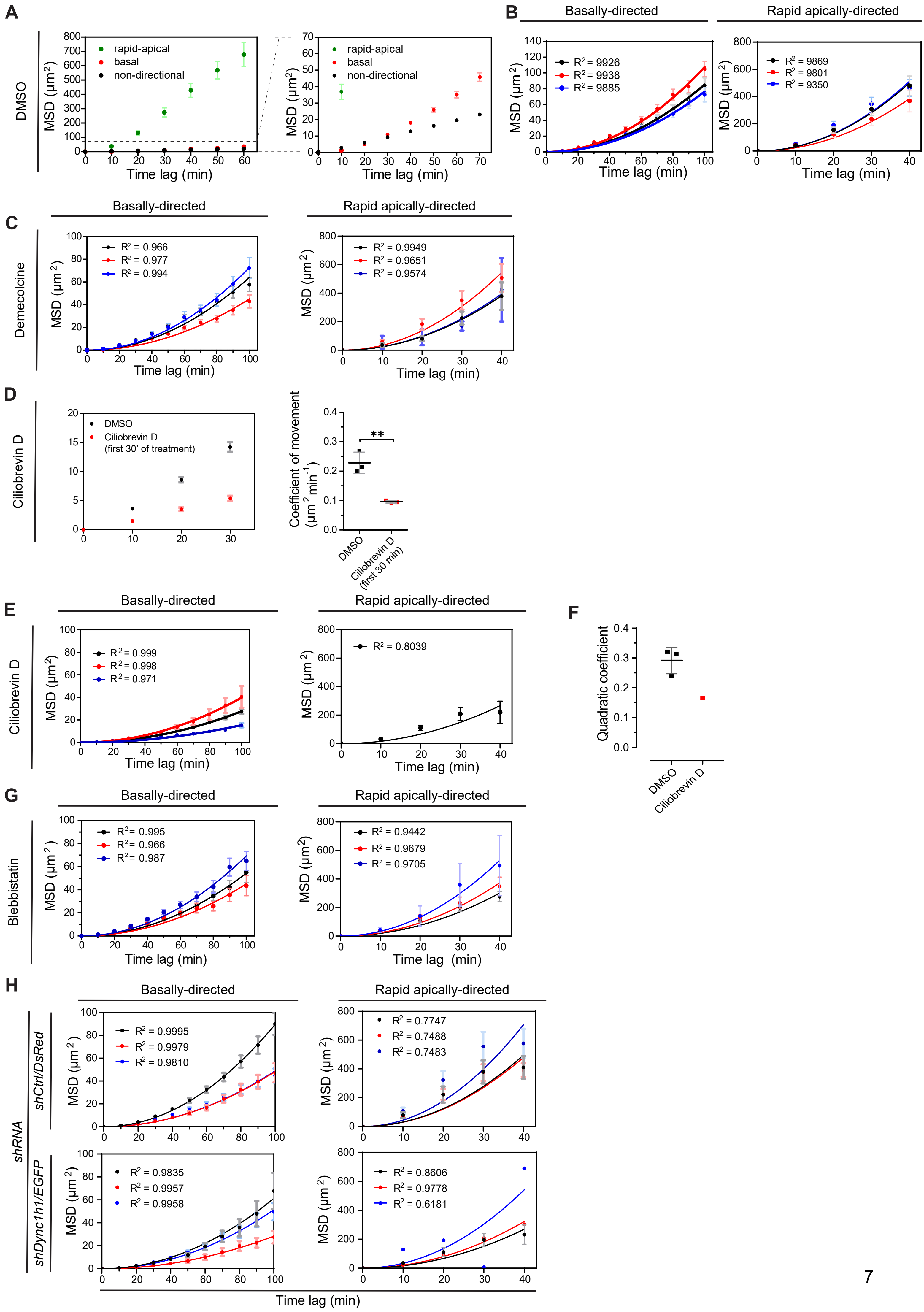
A
DMSO
rapid-apical
basal
non-directional
MSD (μm²)
Time lag (min)
B
Basally-directed
Rapid apically-directed
R² = 9926
R² = 9938
R² = 9885
R² = 9869
R² = 9801
R² = 9350
C
Demecolcine
R² = 0.966
R² = 0.977
R² = 0.994
R² = 0.9949
R² = 0.9651
R² = 0.9574
D
Ciliobrevin D
DMSO
Ciliobrevin D (first 30' of treatment)
Coefficient of movement (μm² min⁻¹)
**
Ciliobrevin D (first 30 min)
E
R² = 0.999
R² = 0.998
R² = 0.971
R² = 0.8039
F
Quadratic coefficient
G
Blebbistatin
R² = 0.995
R² = 0.966
R² = 0.987
R² = 0.9442
R² = 0.9679
R² = 0.9705
H
shRNA
shCtrl/DsRed
shDync1h1/EGFP
R² = 0.9995
R² = 0.9979
R² = 0.9810
R² = 0.7747
R² = 0.7488
R² = 0.7483
R² = 0.9835
R² = 0.9957
R² = 0.9958
R² = 0.8606
R² = 0.9778
R² = 0.6181

Supplementary Fig. S3. Mean squared displacement (MSD) comparison of rapid apical, basal and non-directional rod nuclear migration.

Related to Fig. 1-5.

(A) MSD profiles of total rapid apical, basal and non-directional rod nuclear translocations in P3 retinae. Right panel displays magnified portion of left panel. Data show mean ± SEM. **(B, C, E, G)** MSD profiles of *total* basal (left panels) and *rapid-apical* rod nuclear translocations (right panels) in P3 retinae exposed to DMSO control **(B)**, Demecolcine **(C)**, Ciliobrevin D **(E)**, and Blebbistatin **(G)**. Different MSD profiles (black, red and blue) are associated with the 3 performed independent experimental repeats in each condition. **(D)** MSD profiles of *total* rod nuclear translocations for initial 30 min of Ciliobrevin D treatment compared with DMSO; corresponding coefficients of movement are shown on the right. In (E), a small number of rapid apical nuclear translocations was observed in 1 of the 3 experimental repeats, thus producing only 1 instead of 3 MSD profiles. **(H)** MSD profiles of *total* basal (left panels) and *rapid-apical* rod nuclear translocations (right panels) in *Nrl.Cre*$^{+/-}$ retinae following electroporation with *shCtrl/DsRed* or *shDync1h1/EGFP* at P1 and culturing for 6DIV. MSD profiles for rapid apically- and basally-directed nuclear translocations throughout this figure were curve fitted with the quadratic function $(y = ax^2)$. Data show mean ± SEM for MSD data points, mean ± SD for coefficient of movement and quadratic coefficient plots. Goodness-of-fit was assessed by R^2 analysis and results are shown in each plot; unpaired *t* test; ***p* < 0.01.

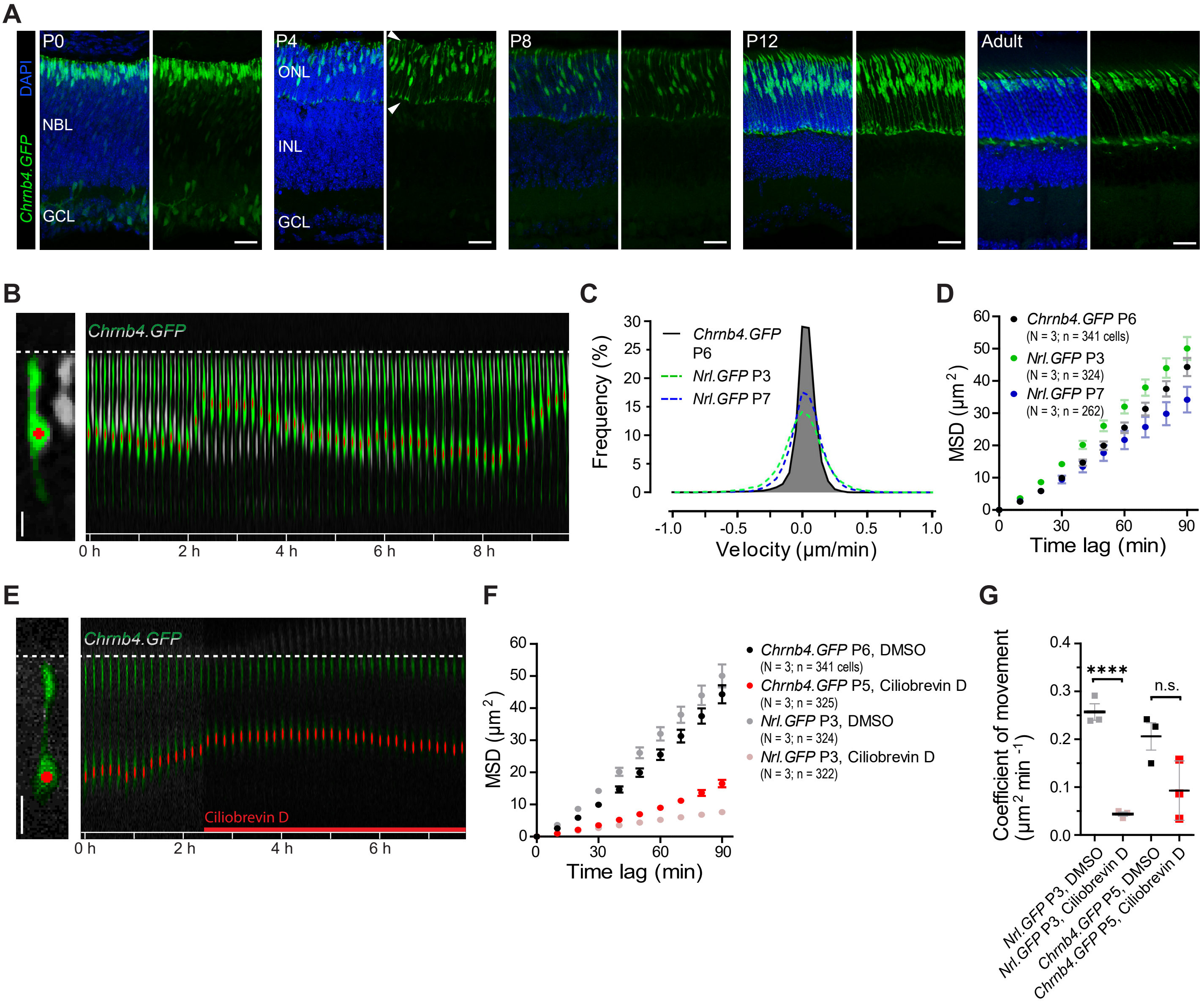
A
DAPI
Chrnb4.GFP
P0
NBL
GCL
P4
ONL
INL
GCL
P8
P12
Adult
B
Chrnb4.GFP
0 h
2 h
4 h
6 h
8 h
C
Frequency (%)
Chrnb4.GFP P6
Nrl.GFP P3
Nrl.GFP P7
Velocity (µm/min)
D
MSD (µm²)
Chrnb4.GFP P6 (N = 3; n = 341 cells)
Nrl.GFP P3 (N = 3; n = 324)
Nrl.GFP P7 (N = 3; n = 262)
Time lag (min)
E
Chrnb4.GFP
Ciliobrevin D
0 h
2 h
4 h
6 h
F
MSD (µm²)
Chrnb4.GFP P6, DMSO (N = 3; n = 341 cells)
Chrnb4.GFP P5, Ciliobrevin D (N = 3; n = 325)
Nrl.GFP P3, DMSO (N = 3; n = 324)
Nrl.GFP P3, Ciliobrevin D (N = 3; n = 322)
Time lag (min)
G
Coefficient of movement (µm² min⁻¹)
****
n.s.
Nrl.GFP P3, DMSO
Nrl.GFP P3, Ciliobrevin D
Chrnb4.GFP P5, DMSO
Chrnb4.GFP P5, Ciliobrevin D

Supplementary Fig. S4. Cone photoreceptors undergo dynein-dependent apico-basal nuclear translocations.

Related to Figs. 1, 4.

(A) *EGFP* expression in the *Chrnb4-EGFP* retina at various developmental stages and in the adult mouse. White arrow heads at P4 indicate apical and basal cone processes. **(B)** Time lapse series of a manually segmented cone (green) nucleus (red dot) migrating in the apico-basal cellular axis in P3 *Chrnb4.EGFP* retina (grayscale). **(C)** Comparison of total cone nuclear velocity distributions at P6 (*Chrnb4.EGFP*) with total rod nuclear velocity distributions at P3 and P7 (*Nrl.GFP*$^{+/+}$). **(D)** MSD profiles of total cone nuclear translocations observed at P6 (*Chrnb4.EGFP*) compared with total rod nuclear velocity distributions at P3 and P7 (*Nrl.GFP*$^{+/+}$). **(E)** Time lapse of a manually segmented cone (green) nucleus (red dot) migrating in the apico-basal cellular axis in a P5 *Chrnb4.EGFP* retina (grayscale) exposed to Ciliobrevin D from 120 min onwards. **(F)** MSD profiles of total cone nuclear translocations in Ciliobrevin D vs DMSO, compared with MSD profiles of total rod nuclear translocations at P3. **(G)** Coefficients of movement for total cone nuclear translocations in Ciliobrevin D vs DMSO and total rod nuclear translocations in P3 retinae. Experimental repeats, each containing a whole set of nuclear trajectories, were collapsed down to individual data points. Scale bars, (**A**) 25 µm, (**B, E**) 5 µm. Unpaired t test; n.s., not significant; ****$p < 0.0001$. Data show mean ± SEM (**D, F**).

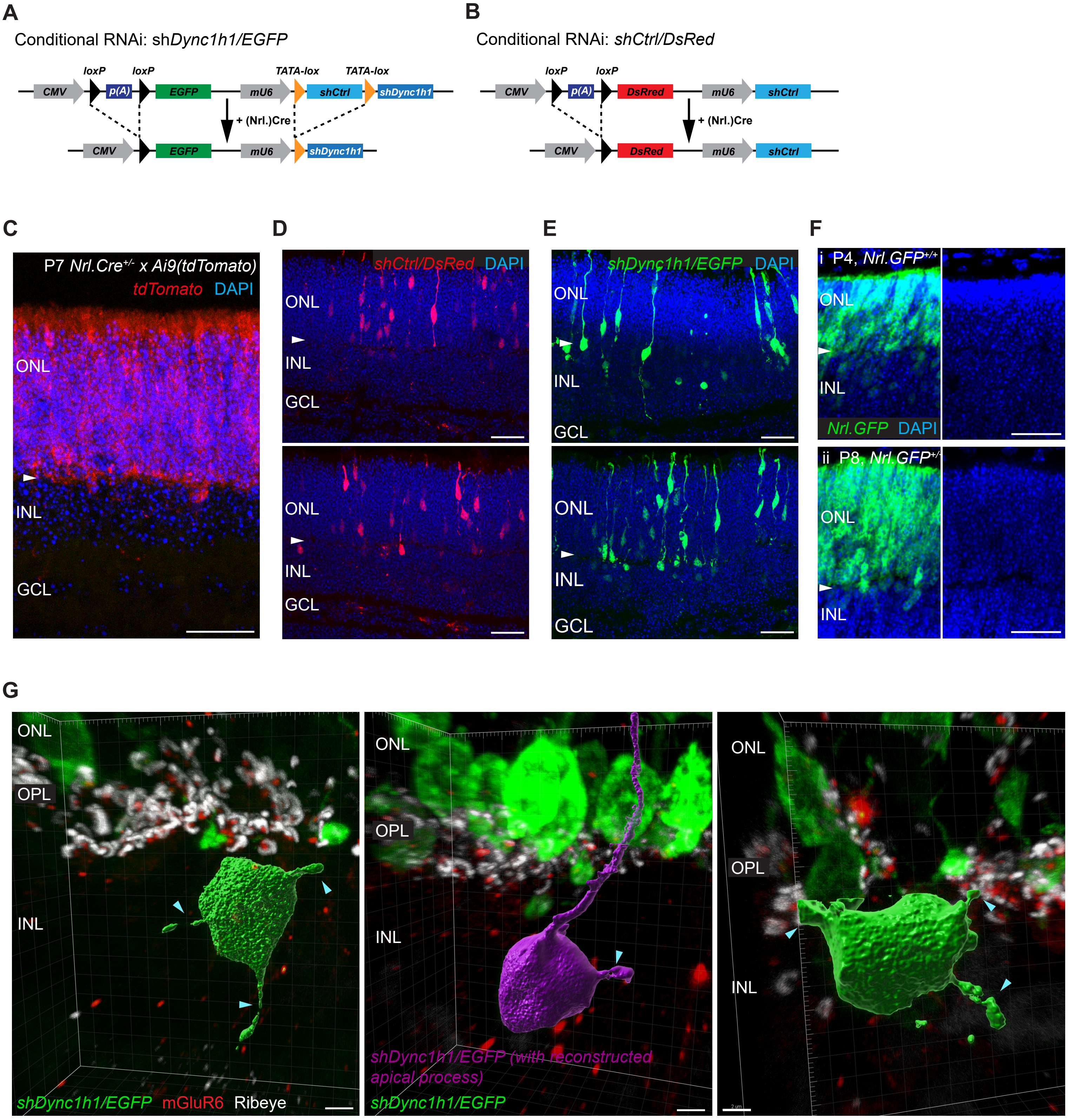
A
Conditional RNAi: shDync1h1/EGFP
loxP
loxP
TATA-lox
TATA-lox
CMV
p(A)
EGFP
mU6
shCtrl
shDync1h1
+ (Nrl.)Cre
B
Conditional RNAi: shCtrl/DsRed
DsRed
C
P7 Nrl.Cre+/- x Ai9(tdTomato)
tdTomato DAPI
ONL
INL
GCL
D
shCtrl/DsRed DAPI
E
shDync1h1/EGFP DAPI
F
i P4, Nrl.GFP+/+
Nrl.GFP DAPI
ii P8, Nrl.GFP+/+
G
OPL
shDync1h1/EGFP mGluR6 Ribeye
shDync1h1/EGFP (with reconstructed apical process)
shDync1h1/EGFP

Supplementary Fig. S5. Conditional dynein 1 loss-of-function in rods results in ectopically located photoreceptors and disrupted ONL lamination.

Related to Figs. 5, 6.

(A) Schematic of *shDync1h1/EGFP* gene silencing construct for conditional Cre-mediated (*Nrl.Cre*$^{+/-}$ mouse) short hairpin (*shDync1h1*) and reporter gene (EGFP) expression in rods. **(B)** Schematic of control construct for conditional Cre-mediated scrambled *shCtrl* short hairpin and *DsRed* expression. **(C)** Cre expression is restricted to rod PRs in P4 *Nrl.Cre*$^{+/-}$ *x Ai9* mice, as previously reported by Brightman et al, 2016 ([55]). The *Ai9* mouse strain is a ubiquitous Cre reporter line, producing tdTomato expression in cells where Cre is expressed.[75] Note that tdTomato-expressing rod cells can be observed in the ONL as well as in ectopic locations (OPL, INL). **(D, E)** Apico-basal positions of transfected rods expressing *shCtrl/DsRed* (**(D)**; red) or *shDync1h1/EGFP* (**(E)**; green) in *Nrl.Cre*$^{+/-}$ retina following electroporation at P1 and culturing for 4DIV. Arrowhead indicates the OPL. Note that basally displaced *shCtrl/DsRed* expressing rods were also be observed (**(D)**, bottom panel), albeit at lower numbers compared with *shDync1h1/EGFP* expressing rods **(E)**. **(F)** Rod PR somata in ectopically basal locations beyond the OPL in the P4 (i) and P8 (ii) *Nrl.GFP*$^{+/+}$ retina. Arrowhead indicates the OPL. **(G)** 3D representations of basally-displaced rod PRs virally transduced with *AAV2/8 shDync1h1/EGFP* (green) at P1 and harvested 3 wks post viral administration. Retinae were immunolabelled for ribeye (grayscale) and mGluR6 (red). Basally-displaced cells frequently exhibit lateral and/or basal processes (cyan arrowheads). Middle panel shows 3D rendered basally displaced shDync1h1/EGFP^{+ve} rod with traceable apical process (magenta). Scale bars, (**C-F**) 25 µm; **(G)** 3 µm.
